# Comparison of *Lactiplantibacillus plantarum* isolates from the gut of mice supplemented with different types of nutrients: a genomic and metabolomic study

**DOI:** 10.3389/fmicb.2023.1295058

**Published:** 2023-11-15

**Authors:** Ziyan Wang, Zhixuan Zhang, Qiuyue Shi, Songyi Liu, Qiaoli Wu, Ze Wang, Emilaguli Saiding, Jiaojiao Han, Jun Zhou, Rixin Wang, Xiurong Su

**Affiliations:** ^1^State Key Laboratory for Managing Biotic and Chemical Threats to the Quality and Safety of Agroproducts, Ningbo, China; ^2^Key Laboratory of Aquacultural Biotechnology Ministry of Education, Ningbo University, Ningbo, China; ^3^School of Marine Sciences, Ningbo University, Ningbo, China

**Keywords:** *Lactiplantibacillus plantarum*, dietary supplements, whole-genome resequencing, intracellular metabolites, environmental adaptability

## Abstract

Many studies have focused on the influence of dietary supplements on gut microbiota composition, but limited research have reported their effects on specific bacterial species in the gut. *Lactiplantibacillus plantarum* is one of the most widely studied probiotics, with a wide range of sources and good environmental adaptability. In this study, in order to elucidate the adaptation strategies of *L. plantarum* to the gut of mice supplemented with carbohydrates, peptides and minerals, whole genome resequencing and intracellular metabolites detection were performed, and high-frequency mutant genes and differential metabolites were screened. The results suggested different types of dietary supplements do have different effects on *L. plantarum* from the gut of mice. Additionally, KEGG annotation unveiled that the effects of these dietary supplements on the gene level of *L. plantarum* primarily pertained to environmental information processing, while the differential metabolites were predominantly associated with metabolism. This study provided new perspectives on the adaptive mechanism of *L. plantarum* in response to the host’s gut environment, suggesting that the diversity of the genome and metabolome of *L. plantarum* was correlated with dietary supplements. Furthermore, this study offered useful guidance in the effective utilization of dietary supplements.

## 1. Introduction

The gut microbiota is an intricate microbial ecosystem which is essential for maintaining gastrointestinal homeostasis, and dysbiosis of them has been linked to the pathogenesis and progression of diverse diseases (Adeshirlarijaney and Gewirtz, 2020; Glassner et al., 2020). Numerous studies have shown that dietary supplements can alleviate disease by improving gut microbiota. For example, supplementation with resveratrol and its derivatives may regulate gut microbiota to improve inflammatory bowel disease (Li M. et al., 2022); supplementation with tuna meat oligopeptides restored gut microbiota homeostasis and improved hyperuricemia (Han et al., 2020b); the gut microbiota also had a significant impact in alleviating pneumonia with high-DHA tuna oil (Chen et al., 2021).

Probiotics are viable microorganisms that, when administered in adequate amounts, promote the well-being of the host in a safe and efficient manner. Among them, *L. plantarum* has gained significant attention in scientific research due to its stable probiotic properties, wide distribution and so on. For probiotic properties, *L. plantarum* LLY-606 has been reported to improve potassium oxonate and hypoxanthine-induced hyperuricemia in mice by regulating intestinal homeostasis and alleviating inflammation (Shi et al., 2023); *L. plantarum* KC3 isolated from fermented kimchi has a respiratory protective effect against inflammation caused by air pollutants (Park et al., 2023); Yogurt-derived *L. plantarum* Q16 played a positive role in alleviating non-alcoholic fatty liver disease induced by a high-fat diet in mice (Tang et al., 2022); In addition, *L. plantarum* JS19 could also improve the disorder of gut microbiota induced by DSS and reduce the manifestation of ulcerative colitis (Ren et al., 2023).

*Lactiplantibacillus plantarum* has a wide range of isolated sources, such as fermented vegetables, dairy products, environmental samples, and the gastrointestinal tracts of mammals and poultry (Siezen et al., 2010), which suggests that *L. plantarum* possesses excellent environmental adaptability. As scientific and technological advancements continue to progress, some high-throughput technologies have been applied to explore the genotype–phenotype correlation of *L. plantarum* at the genetic level to decipher the relationship between bacterial genome composition and ecological niche adaptation, such as comparative genomics and whole-genome resequencing. For example, genomic resequencing and quantitative proteomic analysis revealed the survival mechanism of *L. plantarum* P8 in long-term glucose restriction experiments (He et al., 2018). Resequencing of *L. plantarum* HNU082 isolates was employed to investigate the impact of intestinal selective pressure on its genetic stability in humans, mice, and zebrafish (Huang et al., 2021). In addition, comparative genomics studies on *L. plantarum* from different sources have confirmed that milk-derived and animal-derived isolates have a variety of environment-specific genes (Li K. N. et al., 2022). These studies also indicated that the process of bacterial adaptation to the environment is associated with substantial changes in genomic content, indicating that bacterial genomes reflect the constraints imposed by habitats (Martino et al., 2016).

Few studies have focused on the effects of different dietary supplements on specific strains of bacteria in the host gut. Our study will focus on this, which is based on the following research: Gavage treatment in mice with a dosage of 20 g/kg/day of fermented wax gourd improved gut microbiota and effectively alleviated the impact of *Staphylococcus aureus* on the intestinal tract of mice (Zhang et al., 2021); 50 mg/kg/day *Apostichopus japonicus* oligopeptide administered orally to mice regulated gut microbiota and alleviated hyperuricemia in mice (Lu et al., 2021); Oral gavage of tuna bone powder increased the abundance of anti-inflammatory bacteria and alleviated osteoporosis in mice (Li et al., 2020); Feeding a mixture of 1:1 ratio of basic diet and *Sphacelotheca sorghi* (Link) Clint regulated the structure and diversity of the gut microbiota in mice, and increased the abundance of beneficial bacteria (Li et al., 2021); Oral gavage of sea cucumber active oligopeptides can restore gut microbiota to homeostasis and alleviate fructose induced hyperuricemia in mice (unpublished).

The subjects of this study were isolated from the gastrointestinal tract of mice subjected to different types of nutrient supplements mentioned above. Genome-wide resequencing and intracellular metabolite detection were used to study their differences at the genetic and metabolic levels. To some degree, the findings of this research unveiled the adaptive mechanisms employed by *L. plantarum* in various ecological environments, offering valuable insights for the appropriate utilization of dietary supplements.

## 2. Materials and methods

### 2.1. Isolation, culture, and identification of strains

The nutritional supplementation experiment in mice consisted of five treatments, including oral gavage of fermented wax gourd (Zhang et al., 2021), oral gavage of *Apostichopus japonicus* oligopeptide (Lu et al., 2021), oral gavage of tuna bone powder (Li et al., 2020), feeding a mixture of 1:1 ratio of basic diet and *Sphacelotheca sorghi* (Link) (Li et al., 2021), and oral gavage of sea cucumber peptide. All experimental and animal care procedures were performed according to the Guide for the Care and Use of Laboratory Animals developed by the Ningbo Customs Technology Center, and all of the animal protocols were approved by the Ningbo Customs Technology Center Animal Center. Their license numbers are in order SCXK (ZHE 2014–0001), SYXK (ZHE 2008–0110), SYXK (ZHE 2008–0110), SYXK (ZHE 2018–0003), and SYXK (ZHE 2008–0110). All necessary measures were taken to alleviate pain throughout the experiment.

Fermented wax gourd and *Sphacelotheca sorghi* (Link) are the representatives of carbohydrate; *Apostichopus japonicus* oligopeptide and sea cucumber peptide are the representatives of polypeptides; and tuna bone powder represents minerals. To explore the effects of dietary supplementation with different types of nutrients on *L. plantarum* living in the intestines of mice, the intestinal contents of these mice were collected, respectively, and inoculated with De Man, Rogosa and Sharpe (MRS, Qingdao Hope Bio-Technology Co. Ltd., Qingdao, China) solid medium using a four-zone line method and selectively cultured in an anaerobic gas generation bag (Qingdao Hope Bio-Technology Co. Ltd., Qingdao, China) at 37°C for 24 h. Strains with different morphologies were selected for purification culture (37°C, anaerobic environment, 24 h) until the colonies on solid medium were single morphologies. The consistency of culture conditions, culture medium and culture time was strictly controlled to reduce the influence of *in vitro* cultivation on the characteristics of the strains. Single colonies of the strains were amplified by PCR using 16S rRNA gene primers (27F, 5′- AGA GTT TGA TCC TGG CTC AG-3′, 1492R, 5′- GGT TAC CTT GTT ACG ACT T-3′) (Zhang et al., 2016). Forward sequencing was completed by Sangon Biotech Co., Ltd. (Shanghai, China) and identification was performed via Blast.^1^

### 2.2. Whole-genome resequencing

#### 2.2.1. DNA extraction and construction of the DNA library

One *L. plantarum* was randomly selected from each isolated source according to the results of strain identification. The bacterial precipitation of all five strains of *L. plantarum* was collected after incubation in MRS liquid medium at 37°C overnight without oxygen. After extracting the DNA of these strains (LC-Bio Ltd., Hangzhou, China), the procedure described earlier was employed to construct the DNA library (Ramanathan et al., 2017).

#### 2.2.2. Sequencing, alignment, and annotation

Sequencing was conducted using a HiSeq 4,000 (PE 150) platform in accordance with the instructions. After the whole genome resequencing data were assembled via Megahit (Version 1.1.1), the average nucleotide identity (ANI) was calculated by blast comparison of multiple genomes using OAT software (Lee et al., 2016). ANI is an indicator used to assess the genetic correlation between two genomes at the nucleotide level. It is defined as the average nucleotide similarity between homologous regions of the genomes. ANI is known for its ability to identify closely related species. Genomes with ANI values higher than 95% are considered to belong to the same species (Goris et al., 2007). The junction sequences in reads and low-quality sequencing data were deleted to obtain valid data. The alignment of valid data to the reference genome selected based on Blast and ANI analysis (GCF_003269405.1_ASM326940v1_genomic) was performed using Burrows-Wheeler Aligner (BWA) (Li and Durbin, 2009). The variants were identified via FreeBayes and lumpy-sv, respectively (Garrison and Marth, 2012; Layer et al., 2014), which were annotated with SnpEff software (Cingolani et al., 2012). Finally, to obtain the function of genes, Kyoto Encyclopedia of Genes and Genomes (KEGG)^2^ database and Gene Ontology (GO)^3^ database annotations.

### 2.3. Intracellular metabolite assessment

#### 2.3.1. Harvesting intracellular metabolites

After activation, *L. plantarum* was spread on MRS solid medium and incubated anaerobically overnight at 37°C. The bacteria were collected with 4°C precooled saline and the sediment was washed (8,000 r/min, 10 min) 3 times. Wet bacterial precipitation (0.2 g) from each strain sample was weighed in parallel, with 3 parallel samples in each group. The intracellular metabolites of *L. plantarum* were extracted following the descriptions in a previous study (Ming et al., 2018). Briefly, after freezing and grinding bacteria with liquid nitrogen, the intracellular metabolites was extracted using 5 mL precooled 60% methanol (w/v). After centrifuging the collected liquid at 12000 r/min for 10 min at 4°C, the supernatant was transferred to a centrifuge tube to dry up methanol with flowing nitrogen. When performing derivatization, 100 μL 15 mg/mL methoxylamine hydrochloride/pyridine solution was used to dissolve the precipitate completely and oximate at 37°C for 90 min, and then 100 μL BSTFA (containing 1% TMCS) (Shanghai Aladdin Biochemical Technology Co., Ltd., Shanghai, China) was added and incubated at 70°C for 30 min to complete trimethylsilylation. Subsequently, equal volumes of docosane/n-heptane solution (0.5 mg/mL) were introduced into the derivatives and mixed thoroughly to prepare them for GC–MS analysis.

#### 2.3.2. GC–MS analysis

Derivatives from *L. plantarum* strains were analyzed via a GC–MS system with an Agilent 7,890/M780EI gas chromatograph (GC, Agilent Technologies, Palo Alto, CA, United States) equipped with a fused silica Agilent DB-5MS capillary column (30 m × 0.25 mm × 0.25 μm, Agilent J&W Scientific, Folsom, CA) and a PERSEE mass spectrometer (MS, Shimadzu, Kyoto, Japan). Samples (2 μL) were injected into a DB-5MS capillary column with a split ratio of 8:1 via an Agilent 7683B Series autosampler. In addition, the flow rate of helium as the carrier gas was 1.00 mL/min. The ion source temperature and interface temperature of the mass spectrometer were 250°C and 280°C, respectively. With EI as the ionization mode, the electron energy was set at 70 eV, and the scanning time was 80 min (m/z 45˗550). The temperature program started at 90°C for 3 min, then raised to 160°C at 3°C/min, followed by raising to 220°C at a rate of 2°C/min and maintained for 1 min, and finally raised to 290°C at 3°C/min.

#### 2.3.3. Metabolic data analysis

The mass spectra and retention times were used to identify the metabolites, and their contents were calculated according to the peak areas based on the content of docosane added in the derivative samples. Principal component analysis (PCA) and partial least-squares-discriminant analysis (PLS-DA) were applied to determine the intergroup difference in metabolites. The criteria for identifying differential metabolites among the strains included a significance level of *p* < 0.05 (T test) and VIP ≥ 1. Then the annotation of these metabolites was carried out using the KEGG (see foot note 2) database.

### 2.4. Correlation analysis

The correlation between differential metabolites and genes with high frequency mutations (SNPs>50 or InDels≥5 or SVs ≥ 2) was analyzed by the Pearson method.

### 2.5. Statistical analysis

All metabolic data are expressed as the mean ± standard deviation (SD). For data that conforms to a normal distribution, a student’s *t*-test was carried out to determine the differences between groups. *p* < 0.05 indicated significant differences between groups.

## 3. Results

### 3.1. Strain selection

**Figure 1:**
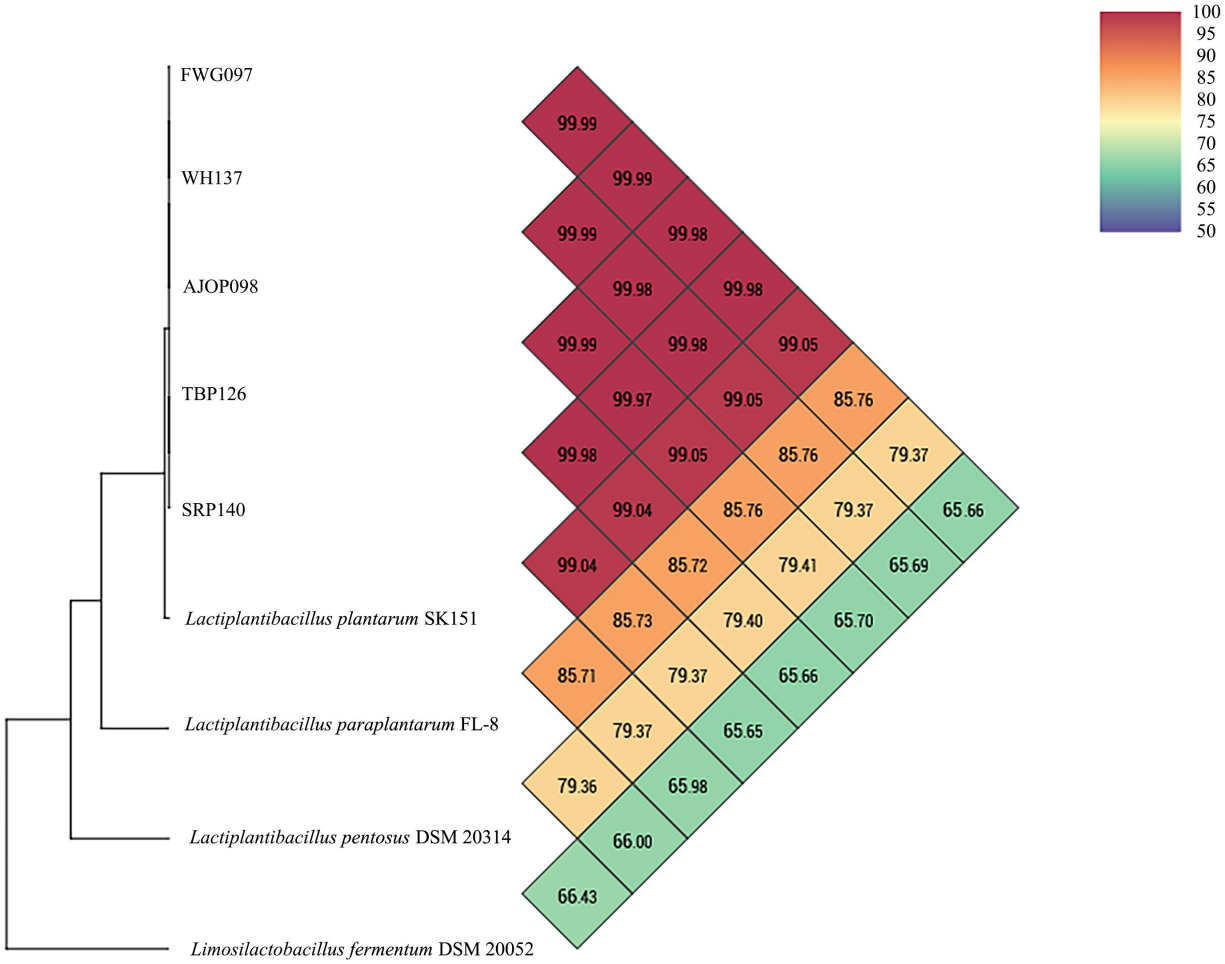
ANI analysis of FWG097, AJOP098, TBP126, WH137, and SRP140 with reference strains.

Based on the strain identification results (Supplementary Figure S1, their accession numbers in GenBank are OR616769–OR616773.), five strains of *L. plantarum* were selected randomly, and the strains were accurately identified by ANI analysis. The results of the ANI analysis showed that FWG097, AJOP098, TBP126, WH137, and SRP140 exhibited OrthoANI values of 99.10, 99.12, 99.11%, 99.12%, and 99.11%, respectively, when compared to the genome of *L. plantarum* SK151. These values were above 95%, indicating a high level of genomic similarity with *L. plantarum* SK151. Furthermore, when compared to the genomes of other closely related strains (the genomes of *L. paraplantarum* FL-8, *L. pentosus* DSM 20314, and *Limosilactobacillus fermentum* DSM 20052 were also used as reference genomes), the OrthoANI values obtained were all in the range of 65.79%–86.33% (Figure 1). Therefore, the selected strains were accurately identified as *L. plantarum*. The brief descriptions of the selected strains are as follows: *L. plantarum* FWG097 was obtained from the gut of mice treated with fermented wax gourd; *L. plantarum* AJOP098 was obtained from the gut of mice that were fed with *Apostichopus japonicus* oligopeptide; *L. plantarum* TBP126 was obtained from the gut of mice treated with tuna bone powder; *L. plantarum* WH137 was obtained from the gut of mice treated with a dominant peptide (GPSGRP) of sea cucumber enzymatic hydrolysate; *L. plantarum* SRP140 was obtained from the gut of mice treated with *Sphacelotheca sorghi* Link.

### 3.2. Genomic features

A total of 6,224,106 reads (0.93 G bases) (*L. plantarum* FWG097), 7,685,518 reads (1.15 G bases) (*L. plantarum* AJOP098), 6,803,464 reads (1.02 G bases) (*L. plantarum* TBP126), 13,662,288 reads (2.05 G bases) (*L. plantarum* WH137), 6,393,238 reads (0.96 G bases) (*L. plantarum* SRP140) were collected from 5 strains of *L. plantarum* via whole-genome resequencing. After aligning the raw data to the reference genome, 6,010,404 reads (0.87G), 7,589,502 reads (1.12G), 6,427,936 reads (0.92G), 13,425,156 reads (1.98G) and 6,214,526 reads (0.91G) valid data were obtained separately, in which data quality ≥Q30 accounted for 93.40, 93.15, 94.15, 94.83 and 93.19%, respectively. Additionally, the GC content and the target coverage depth on average were 45.49% and 263×, respectively. The base coverage rates of the samples are displayed in Supplementary Table S1.

### 3.3. Comparative genomics revealed common SNPs and respective unique SNPs

**Figure 2:**
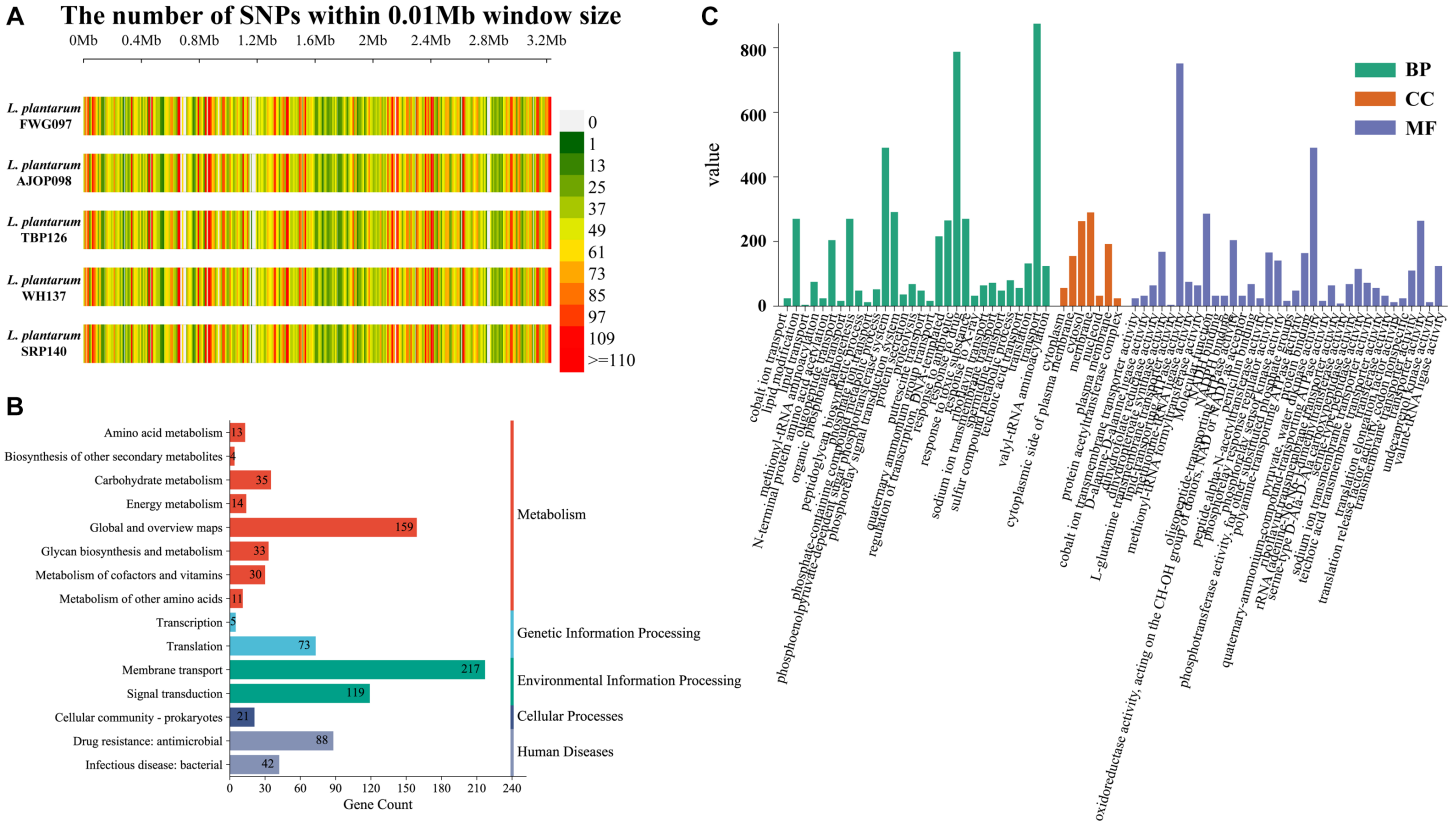
Analysis of SNPs in *L. plantarum*. **(A)** SNP density distribution map of *L. plantarum*. KEGG **(B)** and GO **(C)** annotation of SNPs shared by 5 strains.

Compared with the reference genome, 17,697 (2,361 genes), 17,707 (2,360 genes), 17,702 (2,361 genes), 17,865 (2,363 genes), and 17,728 SNPs (2,360 genes) were detected from 5 strains of *L. plantarum*, respectively, in which 17,511 SNPs distributed over 2,359 genes were shared by the 5 strains. The SNP density map^4^ based on SNPs showed some differences among strains (Figure 2A). Among these SNPs, 7,175 nonsynonymous SNPs were annotated, involving 1,842 genes, which mainly included DNA52_RS01615 (bacterial Ig-like domain-containing protein), DNA52_RS04235 (C40 family peptidase), DNA52_RS05355 (KxYKxGKxW signal peptide domain-containing protein), DNA52_RS11625 (KxYKxGKxW signal peptide domain-containing protein), DNA52_RS04245 (hypothetical protein), and so on (Supplementary Figure S2). KEGG annotation showed that these common genes were related to 15 KEGG pathways. More specifically, the genes were mainly involved in five selected KEGG pathways, membrane transport, global and overview maps, signal transduction, drug resistance: antimicrobial and translation, which accounted for 25.1%, 18.4%, 13.8%, 10.2%, and 8.4% of the total genes, respectively (Figure 2B). GO annotation indicated that these genes involved in 72 GO terms covered three ontologies, biological process (BP, 50.5%), cellular component (CC, 10.3%), and molecular function (MF, 39.2%), which were mainly related to transport (8.9%), response to drug (8.0%), lipid-transporting ATPase activity (7.6%), phosphoenolpyruvate-dependent sugar phosphotransferase system (5.0%) and pyruvate, water dikinase activity (5.0%) (Figure 2C).

In addition to 2 SNPs that had not been annotated, *L. plantarum* FWG097 had 30 unique SNPs, including 13 nonsynonymous SNPs, which were involved in 10 genes. Among them, the genes with more SNPs included DNA52_RS06105 (ATP-binding cassette domain-contain-protein), DNA52_RS11830 (hypothetical protein), DNA52_RS04925 (exonuclease SbcCD subunit D), and DNA52_RS06845 (MarR family transcriptional regulator); as for the 24 SNPs unique to *L. plantarum* AJOP098, 14 nonsynonymous SNPs were annotated, involving 11 genes, which mainly included DNA52_RS00530 (DEAD/DEAH box helicase), DNA52_RS08500 (hypothetical protein) and DNA52_RS11685 (DNA starvation/stationary phase protection protein); excluding SNPs that were not annotated, *L. plantarum* TBP126 had 35 unique SNPs, including 24 nonsynonymous SNPs on 14 genes, in which, DNA52_RS10490 (ABC transporter ATP-binding protein), DNA52_RS02430 (hypothetical protein), DNA52_RS01615 (bacterial Ig-like domain-containing protein), DNA52_RS12690 (DUF1836 domain-containing protein), DNA52_RS11825 (TetR/AcrR family transcriptional regulator), were mainly involved; A total of 95 unique SNPs were annotated for strain *L. plantarum* WH137, including 51 nonsynonymous SNPs, which covered 42 genes, and the genes with a high frequency of SNPs were lepA (elongation factor 4) and DNA52_RS04990 (hypothetical protein); 33 unique SNPs were detected in *L. plantarum* SRP140, of which 23 nonsynonymous SNPs occurred in 13 genes, mainly including DNA52_RS11135 (cadmium resistance transporter), DNA52_RS01615 (bacterial Ig-like domain-containing protein), DNA52_RS03820 (KxYKxGKxW signal peptide domain-containing protein) and DNA52_RS11750 (helix-turn-helix transcriptional regulator) (Supplementary Table S2). Six nonsynonymous SNPs occurred in *L. plantarum* FWG097 and *L. plantarum* WH137 but not in other strains, involving a total of 4 genes (aspartate ammonia-lyase, NAD (P)-dependent alcohol dehydrogenase, DUF805 domain-containing protein and hypothetical protein) (Supplementary Table S3). There were 3 nonsynonymous SNPs that occurred in *L. plantarum* AJOP098 and *L. plantarum* SRP140 but not in other strains (Supplementary Table S4). Two genes encoding hypothetical proteins and 1 gene encoding an ABC-F family ATP-binding cassette domain-containing protein were involved.

### 3.4. Comparative genomics revealed common InDels and respective unique InDels

**Figure 3:**
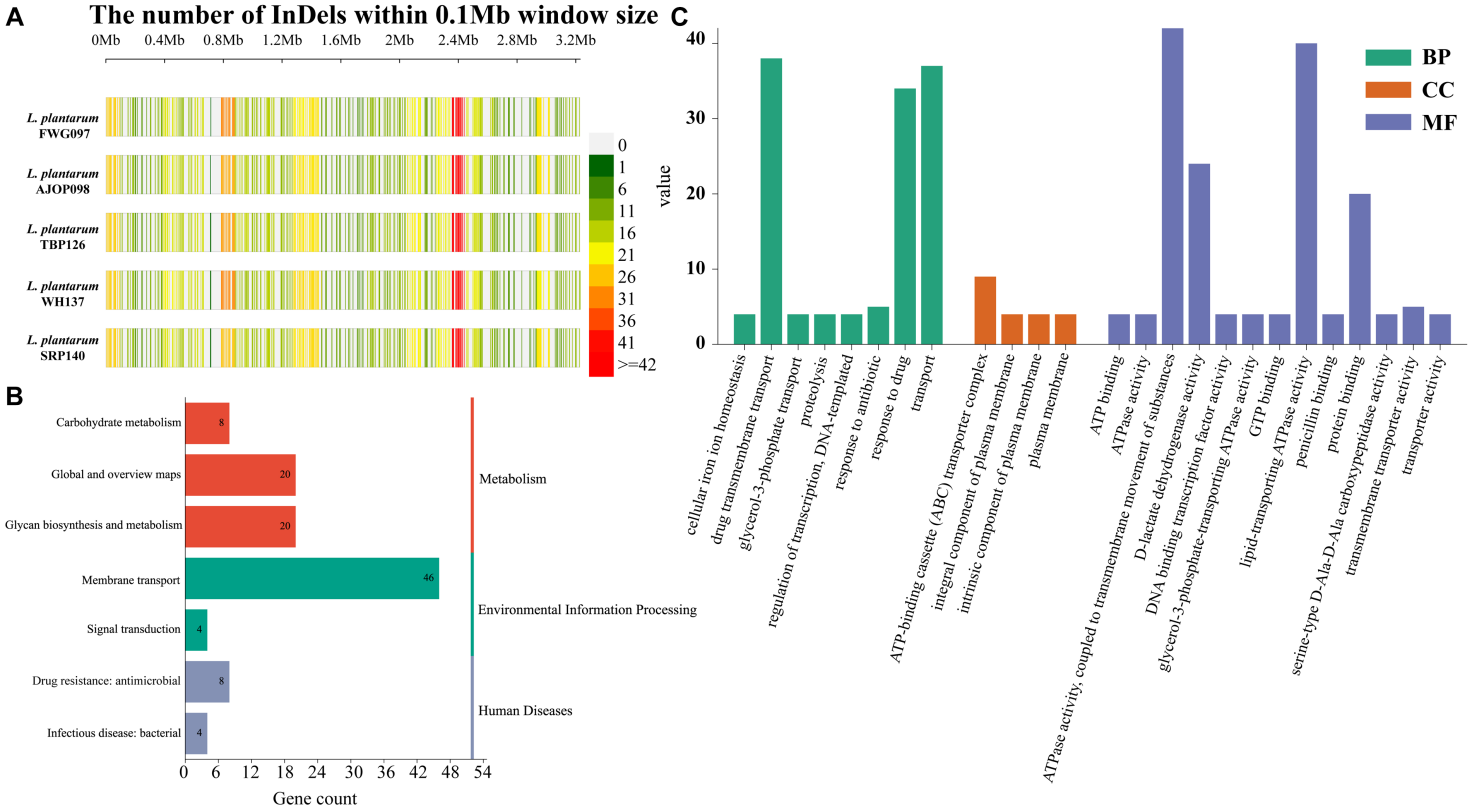
Analysis of InDels in *L. plantarum*. **(A)** InDel density distribution map of *L. plantarum*. KEGG **(B)** and GO **(C)** annotation of InDels shared by 5 strains.

Compared with the reference genome, 506, 507, 514, 518 and 509 InDels were obtained from the 5 strains, respectively (Figure 3A), of which 492 InDels that occurred on 369 genes were shared by 5 strains. DNA52_RS11730 (DUF805 domain-containing protein), DNA52_RS11830 (hypothetical protein), DNA52_RS00100 (hypothetical protein), DNA52_RS04235 (C40 family peptidase) and DNA52_RS11835 (helix-turn-helix domain-containing protein) were the main common genes (Supplementary Figure S2). Functional annotation of these genes presented that these genes were significantly mapped to KEGG pathways related to membrane transport (41.8%), global and overview maps (18.2%) and glycan biosynthesis and metabolism (18.2%) (Figure 3B). In GO annotation, these genes were mainly involved in ATPase activity, coupled to transmembrane movement of substances (13.4%), lipid-transporting ATPase activity (12.7%), drug transmembrane transport (12.1%), and transport (11.8%), which mainly impacted the molecular function ontology (51.9%) (Figure 3C).

There were 4 InDels that were specific to *L. plantarum* FWG097, and these InDels were mapped to 3 genes (2 InDels were detected in DNA52_RS03820 gene encoding KxYKxGKxW signal peptide domain-containing protein); *L. plantarum* AJOP098 did not detect unique InDels; *L. plantarum* TBP126 detected 3 unique InDels, namely, DNA52_RS03820 encoding KxYKxGKxW signal peptide domain-containing protein, DNA52_RS04395 coding for hypothetical protein, and DNA52_RS09270, which coded for NAD (P)-dependent oxidoreductase; 7 unique InDels were detected in *L. plantarum* WH137, involving 5 genes, 2 of which detected 2 InDels (DNA52_RS02205 encoding alpha-l-rhamnosidase and DNA52_RS03820 encoding of KxYKxGKxW signal peptide domain-containing protein); *L. plantarum* SRP140 detected no unique InDels (Supplementary Table S5). There were no InDels detected only in *L. plantarum* FWG097 and *L. plantarum* WH137, but no in other strains. There were also no InDels detected in *L. plantarum* AJOP098 and *L. plantarum* SRP140, but no in other strains.

### 3.5. Comparative genomics revealed common SVs and respective unique SVs

**Figure 4:**
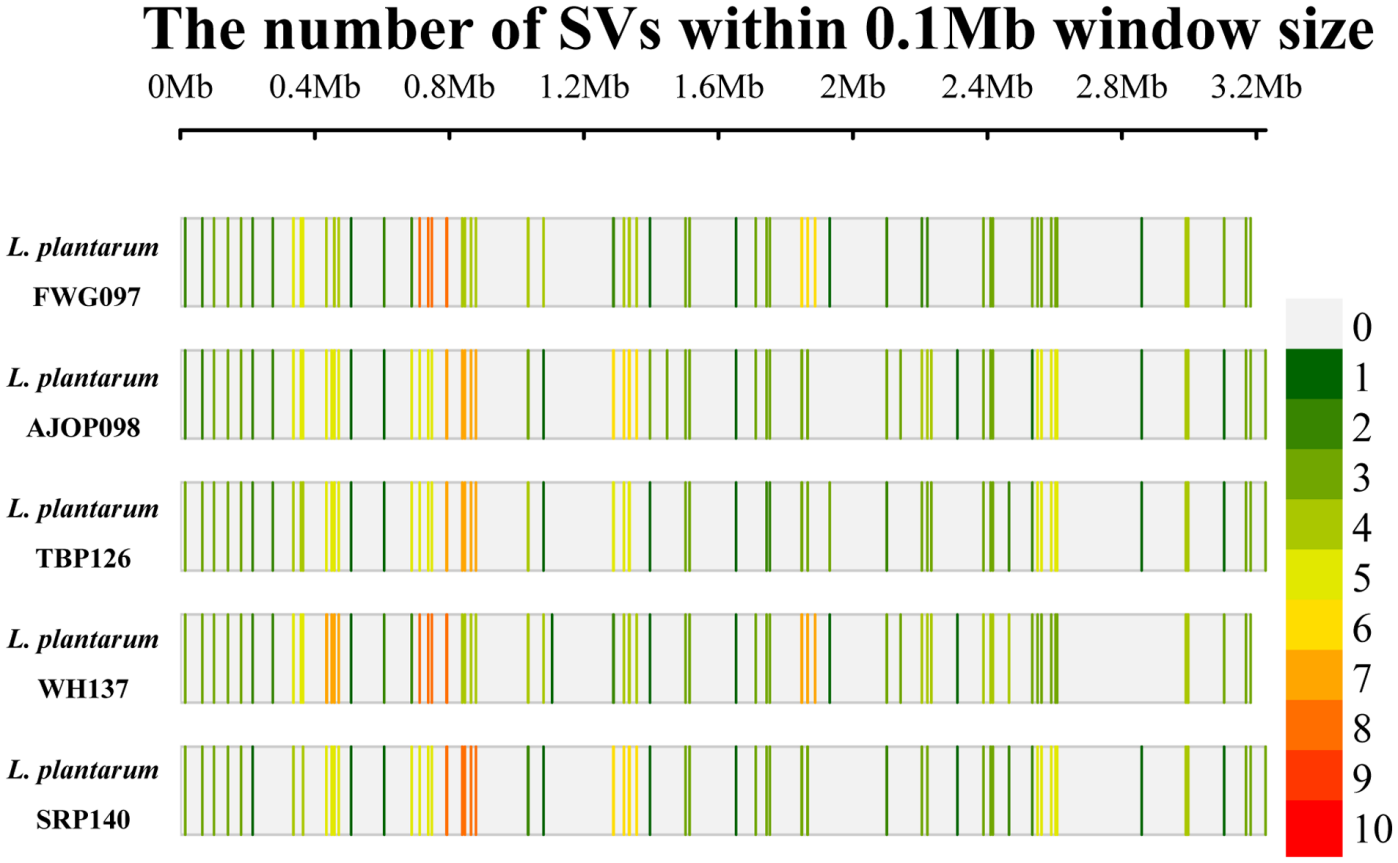
SV density distribution map of *L. plantarum*.

Compared with the reference genome, 77, 83, 78, 87, and 79 SVs were obtained from the 5 strains, respectively (Figure 4), of which 53 SVs were shared by the 5 strains, involving 49 genes. DNA52_RS03820 (KxYKxGKxW signal peptide domain-containing protein), DNA52_RS09185 (GNAT family N-acetyltransferase), and DNA52_RS09270 (NAD (P)-dependent oxidoreductase) were the main common genes. These shared genes were not annotated in the KEGG and GO databases.

Compared with other strains, *L. plantarum* FWG097 had 3 unique SVs, including DNA52_RS02965 (ISL3-like element ISP1 family transposase), DNA52_RS03550 (hypothetical protein) and DNA52_RS12680 (MFS transporter). The 4 unique SVs of *L. plantarum* AJOP098 included DNA52_RS02965 (ISL3-like element ISP1 family transposase), DNA52_RS07120 (HAD family hydrolase), DNA52_RS07115 (fructosamine kinase family protein) and DNA52_RS10840 (cytochrome b5). There were 6 SVs present in *L. plantarum* TBP126 involving 5 genes, namely DNA52_RS03600 (MarR family transcriptional regulator), greA (transcription elongation factor GreA), DNA52_RS04990 (hypothetical protein), rsgA (ribosome small subunit-dependent GTPase A), and DNA52_RS06855 (FAD-dependent oxidoreductase). There were 5 unique SVs in *L. plantarum* WH137, involving DNA52_RS02105 (nitronate monooxygenase), DNA52_RS05355 (KxYKxGKxW signal peptide domain-containing protein), DNA52_RS06515 (hypothetical protein) and DNA52_RS10840 (cytochrome b5) genes. *L. plantarum* SRP140 had no unique annotated SV (Supplementary Table S6). DNA52_RS09385 (LPXTG cell wall anchor domain-containing protein) was the only mutated gene detected in *L. plantarum* FWG097 and *L. plantarum* WH137 but not in the other strains (Supplementary Table S7). There were no SVs that were detected only in *L. plantarum* AJOP098 and *L. plantarum* SRP140.

### 3.6. Comparison of metabolites

**Figure 5:**
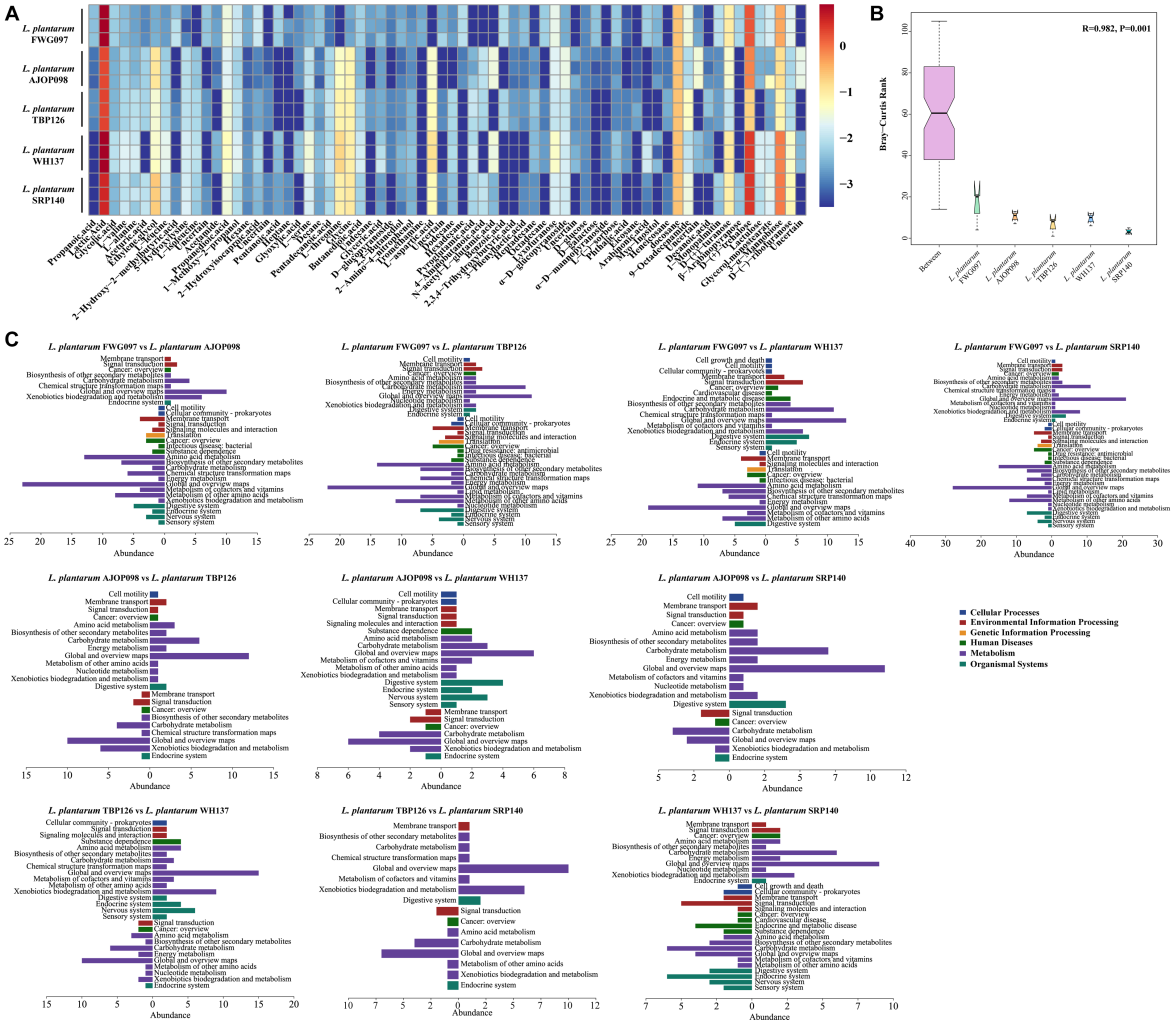
Determination of *L. plantarum* intracellular metabolites. **(A)** Heatmap of the intracellular metabolite contents of *L. plantarum*. **(B)** ANOSIM similarity analysis of intracellular metabolites. **(C)** KEGG annotation of intergroup differential metabolites. Note that the right section represents KEGG pathway analysis of metabolites with higher content in the former sample, and the left section represents KEGG pathway analysis of metabolites with higher content in the latter sample.

A comprehensive analysis using GC–MS identified 70 intracellular metabolites in all, with 6 metabolites classified as uncertain. The total ion chromatography (TIC) can be found in Supplementary Figure S3. The metabolite concentrations were determined by calculating the ratio of peak area to the concentration of the internal standard. All 5 strains of *L. plantarum* produced relatively abundant lactic acid, d-(+)-trehalose, glycine, l-threonine, l-aspartic acid and so on (Figure 5A; Supplementary Table S8). ANOSIM showed significant differences in metabolites among groups, with intergroup differences greater than intragroup differences (Figure 5B).

PLS-DA analysis was used to assist in screening differential metabolites (Supplementary Figure S3; Supplementary Table S9). Compared to *L. plantarum* AJOP098, the content of metabolites like benzoic acid (0.0083 ± 0.0004 μg/mL), 2-hydroxy-2-methylbutyric acid (0.0078 ± 0.003 μg/mL), β-arabinopyranose (0.0065 ± 0.0006 μg/mL), pentanoic acid (0.0057 ± 0.0003 μg/mL), arabinonic acid (0.0054 ± 0.0002 μg/mL), d-glucopyranosid (0.0011 ± 0.0001 μg/mL), and phthalic acid (0.0006 ± 0.0001 μg/mL) in *L. plantarum* FWG097 was relatively high. The metabolites with higher content in *L. plantarum* AJOP098 mainly included l-threonine (0.048 ± 0.0011 μg/mL), l-aspartic acid (0.0406 ± 0.0005 μg/mL), n-acetyl-l-glutamic acid (0.0305 ± 0.0006 μg/mL), 4-aminobutanoic acid (0.01 ± 0.0004 μg/mL), l-isoleucine (0.0045 ± 0.0002 μg/mL), etc. Compared with *L. plantarum L. plantarum* TBP126, the metabolites upregulated by *L. plantarum* FWG097 mainly included d-galactose (0.0043 ± 0.0001 μg/mL), myo-inositol (0.0033 ± 0.0003 μg/mL), glyoxylic acid (0.0014 ± 0.0001 μg/mL), etc., while the metabolites with higher content in *L. plantarum* TBP126 were l-threonine (0.0751 ± 0.0001 μg/mL), l-aspartic acid (0.0574 ± 0.0013 μg/mL), 4-aminobutanoic acid (0.0067 ± 0.0005 μg/mL), l-isoleucine (0.0045 ± 0.0002 μg/mL), pyroglutamic acid (0.0108 ± 0.0021 μg/mL), glycine (0.0731 ± 0.0006 μg/mL) and so on. In the comparison of intracellular metabolites between *L. plantarum* FWG097 and *L. plantarum* WH137, the contents of benzoic acid (0.0083 ± 0.0004 μg/mL), d-galactose (0.0043 ± 0.0001 μg/mL), propanoic acid (0.004 ± 0.0002 μg/mL) and d-glucose (0.0013 ± 0.0001 μg/mL) in *L. plantarum* FWG097 were relatively high. The metabolites with higher concentrations in *L. plantarum* WH137 included l-threonine (0.1661 ± 0.0038 μg/mL), l-aspartic acid (0.1317 ± 0.0025 μg/mL), n-acetyl-l-glutamic acid (0.0308 ± 0.0025 μg/mL), l-isoleucine (0.0095 ± 0.0007 μg/mL) and pyroglutamic acid (0.0112 ± 0.0029 μg/mL). Compared to *L. plantarum* SRP140, the metabolites in *L. plantarum* FWG097 with relatively high concentrations included benzoic acid (0.0083 ± 0.0004 μg/mL), phthalic acid (0.0006 ± 0.0001 μg/mL), d-galactose (0.0043 ± 0.0001 μg/mL), myo-inositol (0.0033 ± 0.0003 μg/mL), and glyoxylic acid (0.0014 ± 0.0001 μg/mL). The metabolites with higher contents in *L. plantarum* SRP140 were l-threonine (0.0824 ± 0.0014 μg/mL), l-aspartic acid (0.0579 ± 0.0011 μg/mL), n-acetyl-l-glutamic acid (0.0031 ± 0.0003 μg/mL), 4-aminobutanoic acid (0.0098 ± 0.0006 μg/mL), l-isoleucine (0.0056 ± 0.0006 μg/mL), glycine (0.0997 ± 0.0005 μg/mL), etc. In the comparison of intracellular metabolites between *L. plantarum* AJOP098 and *L. plantarum* TBP126, *L. plantarum* AJOP098 contained more n-acetyl-l-glutamic acid (0.0305 ± 0.0006 μg/mL), d-galactose (0.0008 ± 0.0003 μg/mL), myo-inositol (0.0013 ± 0.0004 μg/mL), and glyoxylic acid (0.0016 ± 0.0000 μg/mL), while in *L. plantarum* TBP126, benzoic acid (0.0065 ± 0.0001 μg/mL) and phthalic acid (0.0011 ± 0.0000 μg/mL) were more abundant. The contents of 4-aminobutanoic acid (0.0100 ± 0.0004 μg/mL), d-galactose (0.0008 ± 0.0003 μg/mL) and propanoic acid (0.0008 ± 0.0001 μg/mL) in *L. plantarum* AJOP098 were higher than those in *L. plantarum* WH137, while the content of phthalic acid (0.0017 ± 0.0001 μg/mL) in *L. plantarum* WH137 was higher. Compared with *L. plantarum* SRP140, *L. plantarum* AJOP098 contained more d-galactose (0.0008 ± 0.0003 μg/mL), myo-inositol (0.0013 ± 0.0004 μg/mL), glyoxylic acid (0.0016 ± 0.0000 μg/mL), and propanoic acid (0.0008 ± 0.0001 μg/mL). In the comparison between *L. plantarum* TBP126 and *L. plantarum* WH137, the metabolites with higher content in *L. plantarum* TBP126 were mainly 4-aminobutanoic acid (0.0067 ± 0.0005 μg/mL), benzoic acid (0.0065 ± 0.0001 μg/mL) and propanoic acid (0.0014 ± 0.0002 μg/mL). *L. plantarum* WH137 contained more n-acetyl-l-glutamic acid (0.0308 ± 0.0025 μg/mL) and glyoxylic acid (0.0045 ± 0.0000 μg/mL) than the other groups. Compared with *L. plantarum* SRP140, *L. plantarum* TBP126 contained more benzoic acid (0.0065 ± 0.0001 μg/mL), propanoic acid (0.0014 ± 0.0002 μg/mL) and phthalic acid (0.0017 ± 0.0001 μg/mL) than the other groups, while *L. plantarum* SRP140 contained more n-acetyl-l-glutamic acid (0.0031 ± 0.0003 μg/mL). Compared with *L. plantarum* SRP140, *L. plantarum* WH137 had more phthalic acid (0.0017 ± 0.0001 μg/mL) and glyoxylic acid (0.0045 ± 0.0000 μg/mL), while *L. plantarum* SRP140 contained more 4-aminobutanoic acid (0.0098 ± 0.0006 μg/mL) and d-glucose (0.0013 ± 0.0000 μg/mL).

### 3.7. KEGG annotation analysis of differential metabolites

KEGG pathway analysis was conducted on the differential metabolites, and the results can be found in Figure 5C. The upregulated metabolites of *L. plantarum* FWG097 compared with other strains were all mainly related to the global and overview maps, carbohydrate metabolism under the metabolism classification, and membrane transport under the environmental information processing classification of KEGG. Compared with the other strains of *L. plantarum*, the metabolites upregulated in *L. plantarum* AJOP098 mainly involved cell motility under cellular processes, global and overview maps, carbohydrate metabolism, amino acid metabolism under the metabolism category, membrane transport and signal transduction under the environmental information processing classification, and digestive system under the organismal systems classification of KEGG. Metabolites with higher content in *L. plantarum* TBP126 involved a variety of KEGG pathways, mainly including global and overview maps, carbohydrate metabolism and xenobiotics biodegradation and metabolism in metabolism category, membrane transport in environmental information processing classification, and digestive system under organismal systems. The higher content of differential metabolites in *L. plantarum* WH137 was primarily consistent with global and overview maps, amino acid metabolism under metabolism classification, membrane transport in environmental information processing classification, and cancer: overview under human diseases classification. The metabolites significantly upregulated in *L. plantarum* SRP140 were primarily associated with global and overview maps under metabolism, cancer: overview under human diseases classification, signal transduction and amino acid metabolism classification under the environmental information processing classification and endocrine system under organismal systems.

### 3.8. Association analysis

**Figure 6:**
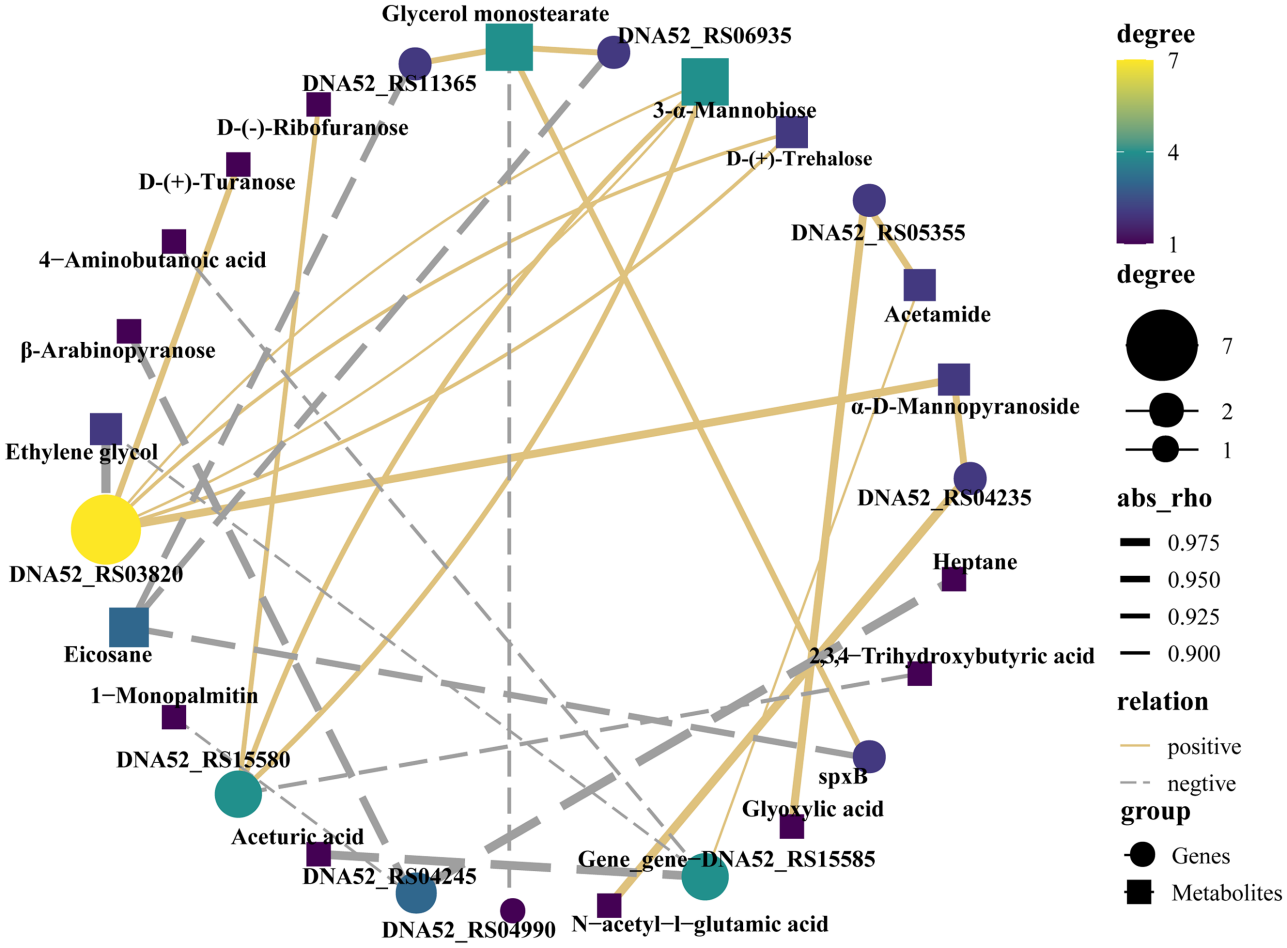
Association analysis of high-frequency mutant genes and differential metabolites.

In the correlation analysis between differential metabolites and high-frequency mutated genes, rho ≥0.5 or rho ≤ −0.5 and *p* < 0.05 were used as screening conditions. The high-frequency mutant gene DNA52_RS03820 is a gene encoding a KxYKxGKxW signal domain-containing protein, that is positively correlated with d-(+)-turanose, 3-α-mannobiose, d-(+)-trehalose and α-d-mannopyranoside and negatively correlated with ethylene glycol in correlation analysis. DNA52_RS15580, encoding a MucBP domain-containing protein, was positively correlated with d-(−)-ribofuranose and 3-α-mannobiose and negatively correlated with 2,3,4-trihydroxybutyric acid. A high-frequency mutant gene, DNA52_RS05355, encoding KxYKxGKxW signal domain-containing protein, was positively correlated with glyoxylic acid and negatively correlated with acetamide. The high-frequency mutated gene DNA52_RS04235 encoding a C40 family peptidase was positively correlated with n-acetyl-l-glutamic acid and α-d-mannopyranoside. SpxB, encoding pyruvate oxidase, was positively correlated with glycerol monostearate but negatively correlated with eicosane (Figure 6).

## 4. Discussion

Studies have demonstrated that during dietary interventions, the gut microbiota of some experimental animals may be changed to a certain extent. For example, tuna roe polypeptides showed antioxidant activity by regulating gut microbiota (Han et al., 2020a), and mannan-oligosaccharide could regulate the gut microbiota and help alleviate metabolic disorders (Wang et al., 2018). In addition, our previous studies have shown that fermented wax gourd and *Sphacelotheca sorghi* (Link) clint could regulate the gut microbiota structure in mice (Li et al., 2021; Zhang et al., 2021). The gut microbiota contributed to the alleviation of hyperuricemia by *Apostichopus japonicus* polypeptide (Lu et al., 2021). The improvement of osteoporosis in mice by tuna bone meal depended on changes in gut microbiota (Li et al., 2020). Aloin can exert the Anti-diabetic effect by regulating gut microbiota and activating JNK-IRS1/PI3K pathway (Zhong et al., 2022). However, the effect of different dietary types on a particular bacterial species in the gut microbiota has rarely been studied.

*Lactiplantibacillus plantarum* is one of the most common probiotics with a variety of probiotic functions, such as alleviating host inflammation (Zhou et al., 2021; Wu et al., 2022) and reducing lipid accumulation (Chen et al., 2023). Moreover, *L. plantarum* has a strong ability to adapt to the environment and will undergo adaptive changes at the gene level when subjected to environmental stress (Song et al., 2018). The five strains of *L. plantarum* in this study were derived from the intestines of mice with different dietary interventions, among which *L. plantarum* FWG097 was obtained from the intestines of mice treated with fermented wax gourd (Zhang et al., 2021), and *L. plantarum* WH137 was obtained from the intestines of mice fed with *Sphacelotheca sorhi* Link (Li et al., 2021). Both dietary supplements had carbohydrates as their main component. Both *L. plantarum* AJOP098 and *L. plantarum* SRP140 were obtained from the intestinal tract of mice fed with dietary peptides (Lu et al., 2021), while *L. plantarum* TBP126 was obtained from the intestines of mice treated with tuna bone meal (Li et al., 2020), which is mainly composed of protein and minerals. Whole-genome resequencing was performed to identify the variation information of each strain, which proved that the 5 strains of *L. plantarum* shared many mutant genes. Among them, the adhesion function of LPXTG cell wall anchor domain-containing protein and MucBP domain-containing protein have been demonstrated (Xu et al., 2022); LysR family transcriptional regulator has been reported to regulate the production of conjugated linoleic acid, which has anti-inflammatory, anticancer, and antidiabetic properties (Liu et al., 2021); 5 strains also share several SNPs that occurred on genes encoding glycosyltransferase, which has been linked to environmental tolerance in *L. brevis* (Fukao et al., 2019); In addition, studies have demonstrated that the presence of exonuclease ABC subunit UvrA plays a role in enhancing the acid adaptation and oxidative stress resistance capabilities of *L. helveticus* (Cappa et al., 2005). The shared mutant genes were primarily associated with the membrane transport (environmental information processing) KEGG pathway. Membrane transport is an important pathway for material exchange between bacteria and their surroundings, and mutations in related genes may be more conducive to the absorption and utilization of nutrients in the intestinal environment.

Some SNPs were detected specifically in strains *L. plantarum* FWG097 and *L. plantarum* WH137, occurring within the coding gene of the aspartate ammonia-lyase, bacterial Ig-like domain-containing protein, NAD (P)-dependent alcohol dehydrogenase and so on. Studies have indicated that the Ig domain have important effect on various processes, including adaptation and adhesion, in vertebrates (Klotz et al., 2020). NAD (P)-dependent alcohol dehydrogenase is involved in the REDOX process of various alcohol compounds, which oxidizes these compounds to the corresponding ketones or aldehydes (Rangel-Porras et al., 2005), providing energy and carbon sources. These genes may be involved in the utilization of carbon sources by *L. plantarum* and regulation of metabolic pathways. There was an SV on the LPXTG cell wall anchor domain-containing protein coding gene detected only in *L. plantarum* FWG097 and *L. plantarum* WH137, and the protein has been proven to have anti-inflammatory function and adhesion properties in *L. reuteri* (Xu et al., 2022). One SNP detected only in *L. plantarum* AJOP098 and *L. plantarum* SRP140 occurred in the ABC-F family ATP-binding cassette domain-containing protein coding gene, which was suspected to be related to pH adaptation in bacteria in previous studies (Msimbira et al., 2022). The SNPS unique to *L. plantarum* TBP126 involved bacterial Ig-like domain-containing protein (Klotz et al., 2020), MarR family transcriptional regulator, TetR/AcrR family transcriptional regulator and ABC transporter ATP-binding protein coding genes. TetR/AcrR family transcriptional regulator and MarR family transcriptional regulator have been demonstrated to help bacteria adapt to the environment and regulate the expression of specific genes in bacteria (Yang et al., 2019; He et al., 2020; Alves et al., 2021). These regulators can either promote or inhibit the absorption, utilization, and metabolic processes of specific minerals, ultimately impacting bacterial metabolism and growth. The ABC transporter ATP-binding protein is a component of the ABC transport system, which forms a functional complex with transmembrane channel proteins (Manjula et al., 2015). This complex plays a role in the active transport of specific minerals, thereby influencing bacterial metabolism and growth. We conjecture that these differences in specific variation may be attributed to different kinds of dietary supplements, but in general, they are conducive to the acclimatization of *L. plantarum* in the habitat, and the potential causal relationship needs further investigation.

The differential metabolite-related KEGG pathways of the five strains were similar, but there were also some differences. For instance, different from *L. plantarum* FWG097, under the primary classification of environmental information processing, the upregulated differential metabolites of *L. plantarum* AJOP098 were also mainly related to signal transduction, and under the metabolism category, amino acid metabolism was the main enrichment pathway. The differential metabolites of *L. plantarum* TBP126 compared with other strains were more concentrated in xenobiotics biodegradation and metabolism pathway under the metabolism primary KEGG classification. These results suggested that *L. plantarum* might rely on different pathways to adapt to different gut environments (Wang et al., 2023). Benzoic acid is a compound with potential antifungal properties, with significantly higher levels in *L. plantarum* FWG097 and *L. plantarum* TBP126 compared to several other strains, which may indicate that supplementation with fermented wax gourd and tuna bone meal can exert antibacterial functions by altering the metabolites of the strains (Lipińska et al., 2018). The content of d-(+) -trehalose in *L. plantarum* TBP126 was relatively low, while more d-(+)-trehalose was produced in other strains. A previous investigation proposed the potential of trehalose as a favorable prebiotic for combating salmonellosis, and dietary supplementation with trehalose can reduce intestinal damage (Wu et al., 2020). L-isoleucine is important for several physiological functions and immune maintenance in humans and animals, and it has also been reported to relieve colitis in rats (Mao et al., 2022). In this study, all strains except *L. plantarum* FWG097 produced higher levels of l-isoleucine, which is beneficial to the intestinal health of the host. Overall, the different dietary supplements in this study did cause differences in the metabolites of *L. plantarum* in the gut, which affect the intestinal environment to some extent.

The MucBP domain-containing protein (DNA52_RS15580) in *Lactobacillus* is responsible for the specific adhesion function of microorganisms in the host (Devi and Halami, 2017). D-(−)-ribofuranose analogs have been reported to have potential analgesic and anti-inflammatory activities (Spriha et al., 2021). 3-Alpha-mannobiose could improve the structure of the gut microbiota and is beneficial to intestinal health. In summary, DNA52_RS15580, d-(−)-ribofuranose, and 3-alpha-mannobiose are beneficial to host health, which confirmed the positive correlation between DNA52_RS15580 and d-(−)-ribofuranose and 3-alpha-mannobiose in this study. DNA52_RS03820 and DNA52_RS05355 encoded KxYKxGKxW signal domain-containing protein was correlated with many different metabolites, such as d-(+)-turanose and d-(+)-trehalose. Previous studies have demonstrated the potential anti-inflammatory effects of turanose and trehalose *in vitro* (Chung et al., 2017; Yu et al., 2023), and trehalose has also been shown to regulate glucose metabolism and maintain homeostasis (Sokołowska et al., 2022). Nevertheless, there is currently limited research on the KxYKxGKxW signal domain-containing protein. Based on the correlation analysis in this study, it is speculated that this protein may have certain probiotic properties, such as anti-inflammatory properties.

## 5. Conclusion

Genome-wide resequencing revealed the diverse influences of different kinds of dietary interventions (carbohydrates, peptides or minerals) on the gene levels of *L. plantarum* in the gut of mice. According to the results of KEGG analysis, their adaptation to the intestinal habitat primarily occurred through pathways associated with processing environmental information. Different types of dietary interventions also had certain effects on the intracellular metabolites of the strains, which were primarily associated with metabolic KEGG pathways. This study elucidated that different dietary supplements exert distinct effects on particular bacteria (*L. plantarum*) in the host gut, which provides valuable insights that can guide the appropriate utilization of dietary nutritional supplements.

## Data availability statement

The datasets presented in this study can be found in online repositories. The names of the repository/repositories and accession number(s) can be found at: https://www.ncbi.nlm.nih.gov/, PRJNA1012525.

## Author contributions

ZiW: Conceptualization, Formal analysis, Investigation, Methodology, Software, Validation, Visualization, Writing – original draft. ZZ: Conceptualization, Formal analysis, Investigation, Methodology, Software, Writing – original draft. QS: Formal analysis, Investigation, Software, Writing – original draft. SL: Formal analysis, Writing – original draft, Validation, Visualization. QW: Formal analysis, Validation, Visualization, Writing – original draft. ZeW: Formal analysis, Validation, Visualization, Writing – original draft. ES: Formal analysis, Validation, Visualization, Writing – original draft. JH: Data curation, Supervision, Writing – review & editing, Investigation. JZ: Data curation, Investigation, Supervision, Writing – review & editing. RW: Data curation, Supervision, Writing – review & editing, Investigation. XS: Conceptualization, Data curation, Project administration, Resources, Supervision, Writing – review & editing.

## Footnotes

^1^ https://blast.ncbi.nlm.nih.gov/Blast.cgi

^2^ https://www.kegg.jp/

^3^ http://geneontology.org/

^4^ https://www.bioinformatics.com.cn
