# Supplementary material for: Comparison of Lactiplantibacillus plantarum isolates from the gut of mice supplemented with different types of nutrients: a genomic and metabolomic study
Source: Front Microbiol. 2023 Nov 15;14:1295058. doi: 10.3389/fmicb.2023.1295058 (PMC10684713; doi:10.3389/fmicb.2023.1295058)
Supplement: Supplementary file 1 [file Data_Sheet_1.docx]

**Table. S1 Base coverage rates of the samples**

| Term | *L. plantarum* FWG097 | *L. plantarum* AJOP098 | *L. plantarum* TBP126 | *L. plantarum* WH137 | *L. plantarum* SRP140 |
| --- | --- | --- | --- | --- | --- |
| Base Coverage Rate | 90.05% | 90.05% | 90.02% | 90.13% | 90.04% |

**Table. S2 SNPs unique to each strain**

| Strains | Gene Name | Gene Description | Value |
| --- | --- | --- | --- |
| *L.* *plantarum* FWG097 | DNA52_RS04925 | exonuclease SbcCD subunit D | 2 |
| *L.* *plantarum* FWG097 | DNA52_RS06845 | MarR family transcriptional regulator | 2 |
| *L.* *plantarum* FWG097 | DNA52_RS11830 | hypothetical protein | 2 |
| *L.* *plantarum* FWG097 | DNA52_RS06105 | ATP-binding cassette domain-containing protein | 1 |
| *L.* *plantarum* FWG097 | celB | PTS cellobiose transporter subunit IIC | 1 |
| *L.* *plantarum* FWG097 | DNA52_RS03430 | MucBP domain-containing protein | 1 |
| *L.* *plantarum* FWG097 | DNA52_RS10030 | ABC transporter permease | 1 |
| *L.* *plantarum* FWG097 | DNA52_RS11730 | DUF805 domain-containing protein | 1 |
| *L.* *plantarum* FWG097 | DNA52_RS12275 | tyrosine-protein phosphatase | 1 |
| *L.* *plantarum* FWG097 | DNA52_RS12940 | GNAT family N-acetyltransferase | 1 |
| *L.* *plantarum* AJOP098 | DNA52_RS00530 | DEAD/DEAH box helicase | 2 |
| *L.* *plantarum* AJOP098 | DNA52_RS08500 | hypothetical protein | 2 |
| *L.* *plantarum* AJOP098 | DNA52_RS11685 | DNA starvation/stationary phase protection protein | 2 |
| *L.* *plantarum* AJOP098 | DNA52_RS02150 | LPXTG cell wall anchor domain-containing protein | 1 |
| *L.* *plantarum* AJOP098 | DNA52_RS08755 | helix-turn-helix transcriptional regulator | 1 |
| *L.* *plantarum* AJOP098 | DNA52_RS02410 | MerR family transcriptional regulator | 1 |
| *L.* *plantarum* AJOP098 | DNA52_RS09535 | bacteriocin immunity protein | 1 |
| *L.* *plantarum* AJOP098 | DNA52_RS10340 | YueI family protein | 1 |
| *L.* *plantarum* AJOP098 | DNA52_RS11785 | SH3 domain-containing protein | 1 |
| *L.* *plantarum* AJOP098 | DNA52_RS14170 | nitroreductase | 1 |
| *L.* *plantarum* AJOP098 | DNA52_RS14335 | proline iminopeptidase-family hydrolase | 1 |
| *L.* *plantarum* TBP126 | xrtG | exosortase family protein XrtG | 5 |
| *L.* *plantarum* TBP126 | DNA52_RS02430 | hypothetical protein | 5 |
| *L.* *plantarum* TBP126 | DNA52_RS01615 | bacterial Ig-like domain-containing protein | 2 |
| *L.* *plantarum* TBP126 | DNA52_RS12690 | DUF1836 domain-containing protein | 2 |
| *L.* *plantarum* TBP126 | DNA52_RS01760 | hypothetical protein | 1 |
| *L.* *plantarum* TBP126 | DNA52_RS07705 | putative glycosyltransferase%2C exosortase G system-associated | 1 |
| *L.* *plantarum* TBP126 | DNA52_RS08080 | competence protein | 1 |
| *L.* *plantarum* TBP126 | DNA52_RS09195 | VOC family protein | 1 |
| *L.* *plantarum* TBP126 | DNA52_RS09270 | NAD(P)-dependent oxidoreductase | 1 |
| *L.* *plantarum* TBP126 | DNA52_RS11825 | TetR/AcrR family transcriptional regulator | 1 |
| *L.* *plantarum* TBP126 | DNA52_RS14590 | ECF-type riboflavin transporter substrate-binding protein | 1 |
| *L.* *plantarum* TBP126 | DNA52_RS08930 | serine hydrolase | 1 |
| *L.* *plantarum* TBP126 | DNA52_RS15720 | hypothetical protein | 1 |
| *L.* *plantarum* TBP126 | DNA52_RS10490 | ABC transporter ATP-binding protein | 1 |
| *L.* *plantarum* WH137 | DNA52_RS02205 | alpha-L-rhamnosidase | 3 |
| *L.* *plantarum* WH137 | DNA52_RS02895 | hypothetical protein | 2 |
| *L.* *plantarum* WH137 | DNA52_RS04990 | hypothetical protein | 2 |
| *L.* *plantarum* WH137 | DNA52_RS08970 | polysaccharide deacetylase family protein | 2 |
| *L.* *plantarum* WH137 | DNA52_RS09535 | bacteriocin immunity protein | 2 |
| *L.* *plantarum* WH137 | DNA52_RS14020 | IS1182 family transposase | 2 |
| *L.* *plantarum* WH137 | DNA52_RS15535 | oxidoreductase | 2 |
| *L.* *plantarum* WH137 | celB | PTS cellobiose transporter subunit IIC | 1 |
| *L.* *plantarum* WH137 | DNA52_RS00745 | peptide MFS transporter | 1 |
| *L.* *plantarum* WH137 | DNA52_RS02965 | ISL3-like element ISP1 family transposase | 1 |
| *L.* *plantarum* WH137 | DNA52_RS03605 | tyrosine-type recombinase/integrase | 1 |
| *L.* *plantarum* WH137 | DNA52_RS03660 | LTA synthase family protein | 1 |
| *L.* *plantarum* WH137 | DNA52_RS04235 | C40 family peptidase | 1 |
| *L.* *plantarum* WH137 | DNA52_RS04915 | UbiA family prenyltransferase | 1 |
| *L.* *plantarum* WH137 | DNA52_RS05010 | penicillin-binding protein 2 | 1 |
| *L.* *plantarum* WH137 | DNA52_RS05890 | hypothetical protein | 1 |
| *L.* *plantarum* WH137 | DNA52_RS06185 | LD-carboxypeptidase | 1 |
| *L.* *plantarum* WH137 | DNA52_RS06405 | NAD(P)-binding domain-containing protein | 1 |
| *L.* *plantarum* WH137 | DNA52_RS07530 | hypothetical protein | 1 |
| *L.* *plantarum* WH137 | DNA52_RS07725 | Firmicu-CTERM sorting domain-containing protein | 1 |
| *L.* *plantarum* WH137 | DNA52_RS07770 | peptide deformylase | 1 |
| *L.* *plantarum* WH137 | DNA52_RS09185 | GNAT family N-acetyltransferase | 1 |
| *L.* *plantarum* WH137 | DNA52_RS09565 | hypothetical protein | 1 |
| *L.* *plantarum* WH137 | DNA52_RS10470 | LysM peptidoglycan-binding domain-containing protein | 1 |
| *L.* *plantarum* WH137 | DNA52_RS10800 | excinuclease ABC subunit UvrA | 1 |
| *L.* *plantarum* WH137 | DNA52_RS10880 | FAD:protein FMN transferase | 1 |
| *L.* *plantarum* WH137 | DNA52_RS11825 | TetR/AcrR family transcriptional regulator | 1 |
| *L.* *plantarum* WH137 | DNA52_RS12430 | VanZ family protein | 1 |
| *L.* *plantarum* WH137 | DNA52_RS12740 | YxeA family protein | 1 |
| *L.* *plantarum* WH137 | DNA52_RS13480 | GHKL domain-containing protein | 1 |
| *L.* *plantarum* WH137 | DNA52_RS13720 | glycoside hydrolase family 92 protein | 1 |
| *L.* *plantarum* WH137 | DNA52_RS13880 | DegV family protein | 1 |
| *L.* *plantarum* WH137 | DNA52_RS14335 | proline iminopeptidase-family hydrolase | 1 |
| *L.* *plantarum* WH137 | DNA52_RS15545 | ribonuclease H | 1 |
| *L.* *plantarum* WH137 | DNA52_RS15725 | hypothetical protein | 1 |
| *L.* *plantarum* WH137 | greA | transcription elongation factor GreA | 1 |
| *L.* *plantarum* WH137 | lepA | elongation factor 4 | 1 |
| *L.* *plantarum* WH137 | lepB | signal peptidase I | 1 |
| *L.* *plantarum* WH137 | mutL | DNA mismatch repair endonuclease MutL | 1 |
| *L.* *plantarum* WH137 | pyk | pyruvate kinase | 1 |
| *L.* *plantarum* WH137 | rsmI | 16S rRNA (cytidine(1402)-2'-O)-methyltransferase | 1 |
| *L.* *plantarum* WH137 | xrtG | exosortase family protein XrtG | 1 |
| *L.* *plantarum* SRP140 | DNA52_RS11135 | cadmium resistance transporter | 5 |
| *L.* *plantarum* SRP140 | DNA52_RS01615 | bacterial Ig-like domain-containing protein | 4 |
| *L.* *plantarum* SRP140 | DNA52_RS03820 | KxYKxGKxW signal peptide domain-containing protein | 3 |
| *L.* *plantarum* SRP140 | DNA52_RS11750 | helix-turn-helix transcriptional regulator | 2 |
| *L.* *plantarum* SRP140 | DNA52_RS01250 | ABC-F family ATP-binding cassette domain-containing protein | 1 |
| *L.* *plantarum* SRP140 | DNA52_RS01925 | hypothetical protein | 1 |
| *L.* *plantarum* SRP140 | DNA52_RS02900 | L-lactate dehydrogenase | 1 |
| *L.* *plantarum* SRP140 | DNA52_RS03905 | glycerophosphoryl diester phosphodiesterase | 1 |
| *L.* *plantarum* SRP140 | DNA52_RS04615 | WxL domain-containing protein | 1 |
| *L.* *plantarum* SRP140 | DNA52_RS07445 | hypothetical protein | 1 |
| *L.* *plantarum* SRP140 | DNA52_RS07725 | Firmicu-CTERM sorting domain-containing protein | 1 |
| *L.* *plantarum* SRP140 | pyk | pyruvate kinase | 1 |
| *L.* *plantarum* SRP140 | radA | DNA repair protein RadA | 1 |

Note, genes that have not been annotated are not listed.

**Table. S3 SNPs only in *L.* *plantarum* FWG097, *L.* *plantarum* WH137**

| Gene_Name | Gene_Description | Value |
| --- | --- | --- |
| DNA52_RS11365 | NAD(P)-dependent alcohol dehydrogenase | 3 |
| DNA52_RS01615 | bacterial Ig-like domain-containing protein | 1 |
| DNA52_RS04565 | YfhO family protein | 1 |
| DNA52_RS05355 | KxYKxGKxW signal peptide domain-containing protein | 1 |
| DNA52_RS10420 | aspartate ammonia-lyase | 1 |
| DNA52_RS11730 | DUF805 domain-containing protein | 1 |
| DNA52_RS12825 | hypothetical protein | 1 |

Note, genes that have not been annotated are not listed.

**Table. S4 SNPs only in *L.* *plantarum* AJOP098, *L.* *plantarum* SRP140**

| Gene_Name | Gene_Description | Value |
| --- | --- | --- |
| DNA52_RS03820 | KxYKxGKxW signal peptide domain-containing protein | 2 |
| DNA52_RS01250 | ABC-F family ATP-binding cassette domain-containing protein | 1 |
| DNA52_RS01760 | hypothetical protein | 1 |
| DNA52_RS08500 | hypothetical protein | 1 |

Note, genes that have not been annotated are not listed.

**Table. S5 InDels unique to each strain**

| Strains | Gene Name | Gene Description | Value |
| --- | --- | --- | --- |
| *L.* *plantarum* FWG097 | DNA52_RS03820 | KxYKxGKxW signal peptide domain-containing protein | 2 |
| *L.* *plantarum* FWG097 | DNA52_RS12825 | hypothetical protein | 1 |
| *L.* *plantarum* FWG097 | DNA52_RS12940 | GNAT family N-acetyltransferase | 1 |
| *L.* *plantarum* TBP126 | DNA52_RS03820 | KxYKxGKxW signal peptide domain-containing protein | 1 |
| *L.* *plantarum* TBP126 | DNA52_RS04395 | hypothetical protein | 1 |
| *L.* *plantarum* TBP126 | DNA52_RS09270 | NAD(P)-dependent oxidoreductase | 1 |
| *L.* *plantarum* WH137 | DNA52_RS02205 | alpha-L-rhamnosidase | 2 |
| *L.* *plantarum* WH137 | DNA52_RS03820 | KxYKxGKxW signal peptide domain-containing protein | 2 |
| *L.* *plantarum* WH137 | DNA52_RS02475 | threonine/serine exporter family protein | 1 |
| *L.* *plantarum* WH137 | DNA52_RS12430 | VanZ family protein | 1 |
| *L.* *plantarum* WH137 | DNA52_RS15595 | hypothetical protein | 1 |

Note, genes that have not been annotated are not listed.

**Table. S6 SVs unique to each strain**

| Strains | Gene Name | Gene Description | Value |
| --- | --- | --- | --- |
| *L.* *plantarum* FWG097 | DNA52_RS02965 | ISL3-like element ISP1 family transposase | 1 |
| *L.* *plantarum* FWG097 | DNA52_RS03550 | hypothetical protein | 1 |
| *L.* *plantarum* FWG097 | DNA52_RS12680 | MFS transporter | 1 |
| *L.* *plantarum* AJOP098 | DNA52_RS02965 | ISL3-like element ISP1 family transposase | 1 |
| *L.* *plantarum* AJOP098 | DNA52_RS07120 | HAD family hydrolase | 1 |
| *L.* *plantarum* AJOP098 | DNA52_RS07115 | fructosamine kinase family protein | 1 |
| *L.* *plantarum* AJOP098 | DNA52_RS10840 | cytochrome b5 | 1 |
| *L.* *plantarum* TBP126 | DNA52_RS03600 | MarR family transcriptional regulator | 2 |
| *L.* *plantarum* TBP126 | greA | transcription elongation factor GreA | 1 |
| *L.* *plantarum* TBP126 | DNA52_RS04990 | hypothetical protein | 1 |
| *L.* *plantarum* TBP126 | rsgA | ribosome small subunit-dependent GTPase A | 1 |
| *L.* *plantarum* TBP126 | DNA52_RS06855 | FAD-dependent oxidoreductase | 1 |
| *L.* *plantarum* WH137 | DNA52_RS02105 | nitronate monooxygenase | 2 |
| *L.* *plantarum* WH137 | DNA52_RS05355 | KxYKxGKxW signal peptide domain-containing protein | 1 |
| *L.* *plantarum* WH137 | DNA52_RS06515 | hypothetical protein | 1 |
| *L.* *plantarum* WH137 | DNA52_RS10840 | cytochrome b5 | 1 |

Note, genes that have not been annotated are not listed.

**Table. S7 SVs only in *L.* *plantarum* FWG097 and *L.* *plantarum* WH137**

| Gene_Name | Gene_Description | Value |
| --- | --- | --- |
| DNA52_RS09385 | LPXTG cell wall anchor domain-containing protein | 1 |

Note, genes that have not been annotated are not listed.

**Table. S8 Metabolites concentrations**

| Metabolite | Formula | Retention Time (min) | Concentration (μg/mL) | | | | |
| --- | --- | --- | --- | --- | --- | --- | --- |
|  |  |  | *L. plantarum* FWG097 | *L. plantarum* AJOP098 | *L. plantarum* TBP126 | *L. plantarum* WH137 | *L. plantarum* SRP140 |
| Propanoic acid | C_3_H_6_O_2_ | 4.975 | 0.0040±0.0002 | 0.0008±0.0001 | 0.0014±0.0002 | 0±0 | 0±0 |
| Lactic Acid | C_3_H_6_O_3_ | 5.323 | 8.3337±0.0390 | 1.9734±0.0117 | 2.1178±0.0055 | 8.0292±0.0619 | 3.7536±0.0078 |
| Glycolic acid | C_2_H_4_O_3_ | 5.536 | 0.0056±0.0002 | 0.0028±0.0001 | 0.0032±0.0000 | 0.0092±0.0004 | 0.0033±0.0001 |
| L-valine | C_5_H_11_NO_2_ | 5.893 | 0.0035±0.0003 | 0.0055±0.0003 | 0.0070±0.0005 | 0.0178±0.0022 | 0.0109±0.0008 |
| L-alanine | C_3_H_7_NO_2_ | 6.095 | 0.0022±0.0001 | 0.0032±0.0004 | 0.0089±0.0004 | 0.0143±0.0016 | 0.0071±0.0031 |
| Aceturic acid | C_4_H_6_NO_3_ | 6.916 | 0.0013±0.0001 | 0.0060±0.0003 | 0.0064±0.0003 | 0±0 | 0.0100±0.0009 |
| Ethylene glycol | C_2_H_6_O_2_ | 7.158 | 0.0026±0.0002 | 0.1130±0.0005 | 0.0375±0.0013 | 0.0450±0.0035 | 0.2175±0.0057 |
| L-leucine | C_6_H_13_NO_2_ | 7.649 | 0.0018±0.0001 | 0.0056±0.0003 | 0.0073±0.0000 | 0.0226±0.0010 | 0.0109±0.0010 |
| 2-Hydroxy-2-methylbutyric acid | C_5_H_10_O_3_ | 7.91 | 0.0078±0.0030 | 0±0 | 0.0012±0.0003 | 0±0 | 0.0016±0.0000 |
| 5-Hydroxylysine | C_6_H_14_N_2_O_3_ | 8.219 | 0.0010±0.0003 | 0.0088±0.0001 | 0.0097±0.0002 | 0.0156±0.0008 | 0.0164±0.0008 |
| L-isoleucine | C_6_H_13_NO_2_ | 8.315 | 0±0 | 0.0045±0.0002 | 0.0071±0.0001 | 0.0095±0.0007 | 0.0056±0.0006 |
| Uncertain | _ | 8.69 | 0.0047±0.0002 | 0±0 | 0.0016±0.0001 | 0±0 | 0.0014±0.0003 |
| Acetamide | C_2_H_5_NO | 8.663 | 0.0021±0.0002 | 0.0010±0.0002 | 0±0 | 0.0029±0.0002 | 0±0 |
| Propanedioic acid | C_3_H_2_O_4_ | 9.232 | 0.0298±0.0008 | 0.0206±0.0001 | 0.0325±0.0012 | 0.0522±0.0017 | 0.0318±0.0006 |
| 1-Methoxy-2-propanol | C_4_H_10_O_2_ | 9.696 | 0.0124±0.0007 | 0.0064±0.0003 | 0.0097±0.0000 | 0.0200±0.0026 | 0.0076±0.0002 |
| Hexane | C_6_H_14_ | 9.947 | 0.0023±0.0002 | 0.0006±0.0001 | 0.0014±0.0001 | 0.0015±0.0001 | 0.0015±0.0002 |
| 2-Hydroxyisocaproic acid | C_6_H_12_O_3_ | 10.275 | 0.0140±0.0004 | 0.0011±0.0001 | 0.0024±0.0001 | 0.007±0.0002 | 0.0035±0.0002 |
| Uncertain | _ | 10.42 | 0±0 | 0.0008±0.0002 | 0.0014±0.0000 | 0±0 | 0.0013±0.0000 |
| Pentanoic acid | C_5_H_10_O_2_ | 10.478 | 0.0057±0.0003 | 0±0 | 0±0 | 0.0037±0.0003 | 0.0021±0.0001 |
| Heptane | C_7_H_16_ | 10.594 | 0.0011±0.0008 | 0±0 | 0±0 | 0±0 | 0.0014±0.0002 |
| Glyoxylic acid | C_2_H_2_O_3_ | 10.603 | 0.0014±0.0001 | 0.0016±0.0000 | 0±0 | 0.0045±0 | 0±0 |
| L-serine | C_3_H_7_NO_3_ | 11.037 | 0.0025±0.0004 | 0.0102±0.0009 | 0.0073±0.0001 | 0.0313±0.0016 | 0.0170±0.0007 |
| Decane | C_10_H_22_ | 11.366 | 0.0045±0.0002 | 0.0010±0.0003 | 0.0021±0.0005 | 0.0035±0.0004 | 0.0022±0.0002 |
| Pentadecanoic acid | C_15_H_30_O_2_ | 12.39 | 0.0009±0 | 0.0070±0.0007 | 0.0101±0.0014 | 0.0112±0.0022 | 0.0074±0.0015 |
| L-threonine | C_4_H_9_NO_3_ | 12.419 | 0±0 | 0.0480±0.0011 | 0.0751±0.0001 | 0.1661±0.0038 | 0.0824±0.0014 |
| Glycine | C_2_H_5_NO_2_ | 12.795 | 0.0023±0.0003 | 0.0691±0.0009 | 0.0731±0.0006 | 0.0822±0.002 | 0.0997±0.0005 |
| Butanedioic acid | C_4_H_6_O_4_ | 13.268 | 0.0057±0.0001 | 0.0035±0.0002 | 0.0063±0 | 0.0144±0.0018 | 0.0058±0.0002 |
| Undecane | C_11_H_24_ | 13.577 | 0.0019±0.0003 | 0±0 | 0.0019±0 | 0±0 | 0±0 |
| Glyceric acid | C_3_H_6_O_4_ | 13.895 | 0.0031±0.0010 | 0.0020±0.0001 | 0.0024±0.0001 | 0.0090±0.0035 | 0.0020±0.0003 |
| D-glucopyranoside | C_12_H_24_O_6_ | 14.858 | 0.0011±0.0001 | 0±0 | 0.0012±0.0001 | 0.0011±0 | 0±0 |
| 2,3-Butanediol | C_4_H_10_O_2_ | 15.604 | 0.0029±0.0005 | 0.0009±0.0001 | 0.0015±0.0001 | 0.0028±0.0003 | 0.0015±0.0004 |
| 2-Amino-4-nitrophenol | C_6_H_6_N_2_O_3_ | 15.855 | 0.0015±0.0003 | 0.0015±0.0001 | 0.0030±0 | 0.0038±0.0003 | 0.0031±0.0003 |
| Tromethamine | C_4_H_11_NO_3_ | 17.052 | 0.0025±0.0004 | 0.0003±0 | 0±0 | 0±0 | 0±0 |
| L-aspartic acid | C_4_H_7_NO_4_ | 17.448 | 0±0 | 0.0406±0.0005 | 0.0574±0.0013 | 0.1317±0.0025 | 0.0579±0.0011 |
| Uncertain | _ | 18.944 | 0.0040±0.0018 | 0±0 | 0.0013±0 | 0±0 | 0.0011±0.0002 |
| Dodecane | C_12_H_26_ | 19.823 | 0.0018±0.0003 | 0±0 | 0.0071±0.0003 | 0.0070±0.0007 | 0.0038±0.0002 |
| Hexadecane | C_16_H_34_ | 19.977 | 0.0088±0.0005 | 0.0013±0.0002 | 0±0 | 0±0 | 0±0 |
| Pyroglutamic acid | C_5_H_6_NO_3_ | 20.304 | 0±0 | 0.0033±0.0010 | 0.0108±0.0021 | 0.0112±0.0029 | 0.0066±0.0002 |
| 4-Aminobutanoic acid | C_4_H_9_NO_2_ | 21.443 | 0±0 | 0.0100±0.0004 | 0.0067±0.0005 | 0±0 | 0.0098±0.0006 |
| N-acetyl-l-glutamic acid | C_7_H_9_NO_5_ | 21.675 | 0±0 | 0.0305±0.0006 | 0±0 | 0.0308±0.0025 | 0.0031±0.0003 |
| Benzoic acid | C_7_H_6_O_2_ | 21.714 | 0.0083±0.0004 | 0±0 | 0.0065±0.0001 | 0±0 | 0±0 |
| 2,3,4-Trihydroxybutyric acid | C_4_H_8_O_5_ | 22.881 | 0.0018±0 | 0.0010±0.0001 | 0.0021±0.0002 | 0±0 | 0±0 |
| 3-Phenyllactic acid | C_9_H_10_O_3_ | 23.364 | 0.0036±0.0006 | 0.0010±0 | 0.0016±0.0001 | 0±0 | 0.0014±0.0001 |
| Heptadecane | C_17_H_36_ | 27.736 | 0.0004±0.0000 | 0.0018±0.0002 | 0.0042±0.0006 | 0.0036±0.0003 | 0.0017±0.0001 |
| Octadecane | C_18_H_38_ | 27.861 | 0.0057±0.0003 | 0±0 | 0.0012±0.0001 | 0.0019±0.0000 | 0±0 |
| Lyxofuranose | C_13_H_18_O_9_ | 30.67 | 0.0249±0.0008 | 0.0202±0.0002 | 0.0112±0.0004 | 0.0452±0.0007 | 0.0165±0.0002 |
| α-D-glucopyranoside | C_28_H_34_O_15_ | 33.006 | 0.0026±0.0001 | 0.0263±0.0003 | 0.0104±0.0001 | 0.0154±0.0014 | 0.0144±0.0007 |
| Uncertain | _ | 34.29 | 0±0 | 0.0021±0.0009 | 0.0015±0.0002 | 0.0028±0.0006 | 0±0 |
| D-glucose | C_6_H_12_O_6_ | 35.794 | 0.0013±0.0001 | 0.0012±0.0001 | 0.0012±0.0001 | 0.0014±0.0001 | 0.0013±0.0000 |
| D-galactose | C_6_H_12_O_6_ | 35.805 | 0.0043±0.0001 | 0.0008±0.0003 | 0±0 | 0±0 | 0±0 |
| α-D-mannopyranoside | C_12_H_15_NO_8_ | 36.055 | 0±0 | 0.0007±0.0001 | 0±0 | 0.0012±0 | 0±0 |
| L-(-)-sorbose | C_6_H_12_O_6_ | 37.579 | 0.0054±0.0001 | 0.0016±0.0004 | 0.0012±0.0000 | 0.0043±0.0004 | 0.0014±0.0001 |
| Phthalic acid | C_8_H_4_O_4_ | 37.82 | 0.0006±0.0001 | 0±0 | 0.0011±0.0000 | 0.0017±0.0001 | 0±0 |
| Eicosane | C_20_H_42_ | 38.035 | 0±0 | 0.0006±0 | 0.0019±0.0004 | 0±0 | 0±0 |
| Arabinonic acid | C_5_H_10_O_6_ | 42.983 | 0.0054±0.0002 | 0±0 | 0±0 | 0.0129±0.0009 | 0.0012±0.0004 |
| Myo-Inositol | C_6_H_12_O_6_ | 43.572 | 0.0033±0.0003 | 0.0013±0.0004 | 0±0 | 0.0023±0.0012 | 0±0 |
| Heneicosane | C_21_H_44_ | 44.974 | 0±0 | 0.0010±0.0001 | 0.0022±0.0003 | 0±0 | 0.0010±0.0000 |
| Docosane | C_22_H_46_ | 48.591 | 0.25 | 0.25 | 0.25 | 0.25 | 0.25 |
| 9-Octadecenamide | C_18_H_35_NO | 54.402 | 0.0299±0.0017 | 0.0081±0.0006 | 0.0490±0.0035 | 0.0103±0.0015 | 0.0308±0.0007 |
| Uncertain | _ | 59.546 | 0.0017±0.0005 | 0.0347±0.0003 | 0±0 | 0.0035±0.0003 | 0±0 |
| Decanoic acid | C_10_H_20_O_2_ | 62.394 | 0±0 | 0.0013±0.0003 | 0.0034±0.0013 | 0.0022±0.0004 | 0±0 |
| 1-Monopalmitin | C_19_H_38_O_4_ | 63.291 | 0.0093±0.0004 | 0.0015±0.0000 | 0.0020±0 | 0±0 | 0.005±0.0009 |
| D-(+)-turanose | C_12_H_22_O_11_ | 64.159 | 0.0914±0.0067 | 0.0415±0.0018 | 0.0108±0.0003 | 0.1044±0.0027 | 0.0851±0.0007 |
| β-Arabinopyranose | C_5_H_10_O_5_ | 66.737 | 0.0065±0.0006 | 0±0 | 0±0 | 0±0 | 0.0043±0.0005 |
| D-(+)-trehalose | C_12_H_22_O_11_ | 67.741 | 1.6645±0.0039 | 0.8096±0.0021 | 0.1866±0.0005 | 2.4060±0.0138 | 2.8225±0.0037 |
| Lactulose | C_12_H_22_O_11_ | 68.088 | 0.0075±0.0009 | 0.0030±0.0005 | 0±0 | 0.0180±0.0017 | 0.0095±0.0011 |
| Glycerol monostearate | C_21_H_24_O_4_ | 68.822 | 0.0151±0.0004 | 0.0186±0.0225 | 0.0023±0.0002 | 0.0148±0.0008 | 0.0205±0.0014 |
| 3-α-Mannobiose | C_12_H_22_O_11_ | 70.782 | 0.4372±0.0063 | 0.1452±0.0005 | 0.0716±0.0012 | 0.8348±0.0114 | 0.8866±0.0093 |
| D-(-)-ribofuranose | C_5_H_10_O_5_ | 73.687 | 0.0217±0.0011 | 0.0074±0.0006 | 0.0077±0.0000 | 0.0432±0.0027 | 0.0641±0.0008 |
| Uncertain | _ | 74.121 | 0±0 | 0.0260±0.0003 | 0±0 | 0.0033±0.0002 | 0±0 |

**Table. S9 Differential metabolites**

| Metabolites | *L. plantarum* FWG097 vs *L. plantarum* AJOP098 | *L. plantarum* FWG097 vs *L. plantarum* TBP126 | *L. plantarum* FWG097 vs *L. plantarum* WH137 | *L. plantarum* FWG097 vs *L. plantarum* SRP140 | *L. plantarum* AJOP098 vs *L. plantarum* TBP126 | *L. plantarum* AJOP098 vs *L. plantarum* WH137 | *L. plantarum* AJOP098 vs *L. plantarum* SRP140 | *L. plantarum* TBP126 vs *L. plantarum* WH137 | *L. plantarum* TBP126 vs *L. plantarum* SRP140 | *L. plantarum* WH137 vs *L. plantarum* SRP140 |
| --- | --- | --- | --- | --- | --- | --- | --- | --- | --- | --- |
| L-threonine | 🡫 | 🡫 | 🡫 | 🡫 | _ | _ | _ | _ | _ | _ |
| L-aspartic acid | 🡫 | 🡫 | 🡫 | 🡫 | _ | _ | _ | _ | _ | _ |
| N-acetyl-l-glutamic acid | 🡫 | _ | 🡫 | 🡫 | 🡩 | _ | _ | 🡫 | 🡫 | _ |
| 4-Aminobutanoic acid | 🡫 | 🡫 | _ | 🡫 | _ | 🡩 | _ | 🡩 | _ | 🡫 |
| Benzoic acid | 🡩 | _ | 🡩 | 🡩 | 🡫 | _ | _ | 🡩 | 🡩 | _ |
| 2-Hydroxy-2-methylbutyric acid | 🡩 | _ | 🡩 | _ | 🡫 | _ | 🡫 | 🡩 | _ | 🡫 |
| β-Arabinopyranose | 🡩 | 🡩 | 🡩 | _ | _ | _ | 🡫 | _ | 🡫 | 🡫 |
| Octadecane | 🡩 | _ | _ | 🡩 | 🡫 | 🡫 | _ | _ | 🡩 | 🡩 |
| Pentanoic acid | 🡩 | 🡩 | _ | _ | _ | 🡫 | 🡫 | 🡫 | 🡫 | _ |
| Arabinonic acid | 🡩 | 🡩 | _ | _ | _ | 🡫 | 🡫 | 🡫 | 🡫 | _ |
| L-isoleucine | 🡫 | 🡫 | 🡫 | 🡫 | _ | _ | _ | _ | _ | _ |
| Pyroglutamic acid | 🡫 | 🡫 | 🡫 | 🡫 | _ | _ | _ | _ | _ | _ |
| Undecane | 🡩 | _ | _ | _ | 🡫 | _ | _ | 🡩 | 🡩 | _ |
| Dodecane | 🡩 | _ | _ | _ | 🡩 | 🡫 | 🡫 | _ | _ | _ |
| Decanoic acid | 🡫 | 🡫 | 🡫 | _ | _ | _ | 🡩 | _ | 🡩 | 🡩 |
| D-glucopyranoside | 🡩 | _ | _ | 🡩 | 🡫 | 🡫 | _ | _ | 🡩 | 🡩 |
| Heneicosane | 🡫 | 🡫 | _ | 🡫 | _ | 🡩 | _ | 🡩 | _ | 🡫 |
| α-D-mannopyranoside | 🡫 | _ | 🡫 |  | 🡩 | _ | 🡩 | 🡫 | _ | 🡩 |
| Ethylene glycol | 🡫 | _ | _ | 🡫 | _ | _ | _ | _ | _ | _ |
| Eicosane | 🡫 | 🡫 | _ | _ | _ | 🡩 | 🡩 | 🡩 | 🡩 | _ |
| Phthalic acid | 🡩 | _ | _ | 🡩 | 🡫 | 🡫 | _ | _ | 🡫 | 🡩 |
| Hexadecane | _ | 🡩 | 🡩 | 🡩 | 🡩 | 🡩 | 🡩 | _ | _ | _ |
| Lactulose | _ | 🡩 | _ | _ | 🡩 | _ | _ | 🡫 | 🡫 | _ |
| D-galactose | _ | 🡩 | 🡩 | 🡩 | 🡩 | 🡩 | 🡩 | _ | _ | _ |
| Myo-Inositol | _ | 🡩 | _ | 🡩 | 🡩 | _ | 🡩 | _ | _ | _ |
| Tromethamine | _ | 🡩 | 🡩 | 🡩 | 🡩 | 🡩 | 🡩 | _ | _ | _ |
| Acetamide | _ | 🡩 | _ | 🡩 | 🡩 | _ | 🡩 | 🡫 | _ | 🡩 |
| Glyoxylic acid | _ | 🡩 | _ | 🡩 | 🡩 | _ | 🡩 | 🡫 | _ | 🡩 |
| Glycine | _ | 🡫 | _ | 🡫 | _ | _ | _ | _ | _ | _ |
| 1-Monopalmitin | _ | _ | 🡩 | _ | _ | 🡩 | _ | 🡩 | _ | 🡫 |
| Propanoic acid | _ | _ | 🡩 | 🡩 | _ | 🡩 | 🡩 | 🡩 | 🡩 | _ |
| 3-Phenyllactic acid | _ | _ | 🡩 | _ | _ | 🡩 | _ | 🡩 | _ | 🡫 |
| D-glucose | _ | _ | 🡩 | _ | _ | _ | _ | _ | _ | 🡫 |
| 2,3,4-Trihydroxybutyric acid | _ | _ | 🡩 | 🡩 | _ | 🡩 | 🡩 | 🡩 | 🡩 | _ |
| Aceturic acid | _ | _ | 🡩 | _ | _ | 🡩 | _ | 🡩 | _ | 🡫 |
| Heptane | _ | _ | _ | _ | _ | _ | 🡫 | _ | 🡫 | 🡫 |
| D-(+)-trehalose | _ | _ | _ | _ | _ | _ | _ | _ | 🡫 | _ |
| 3-α-Mannobiose | _ | _ | _ | _ | _ | _ | _ | _ | 🡫 | _ |
| Glycerol monostearate | _ | _ | _ | _ | _ | _ | _ | _ | 🡫 | _ |
| D-(-)-ribofuranose | _ | _ | _ | _ | _ | _ | _ | _ | 🡫 | _ |
| D-(+)-turanose | _ | _ | _ | _ | _ | _ | _ | _ | 🡫 | _ |

Note, 🡩 means that a metabolite is more abundant in the former than the latter, and 🡫 represents the opposite trend.


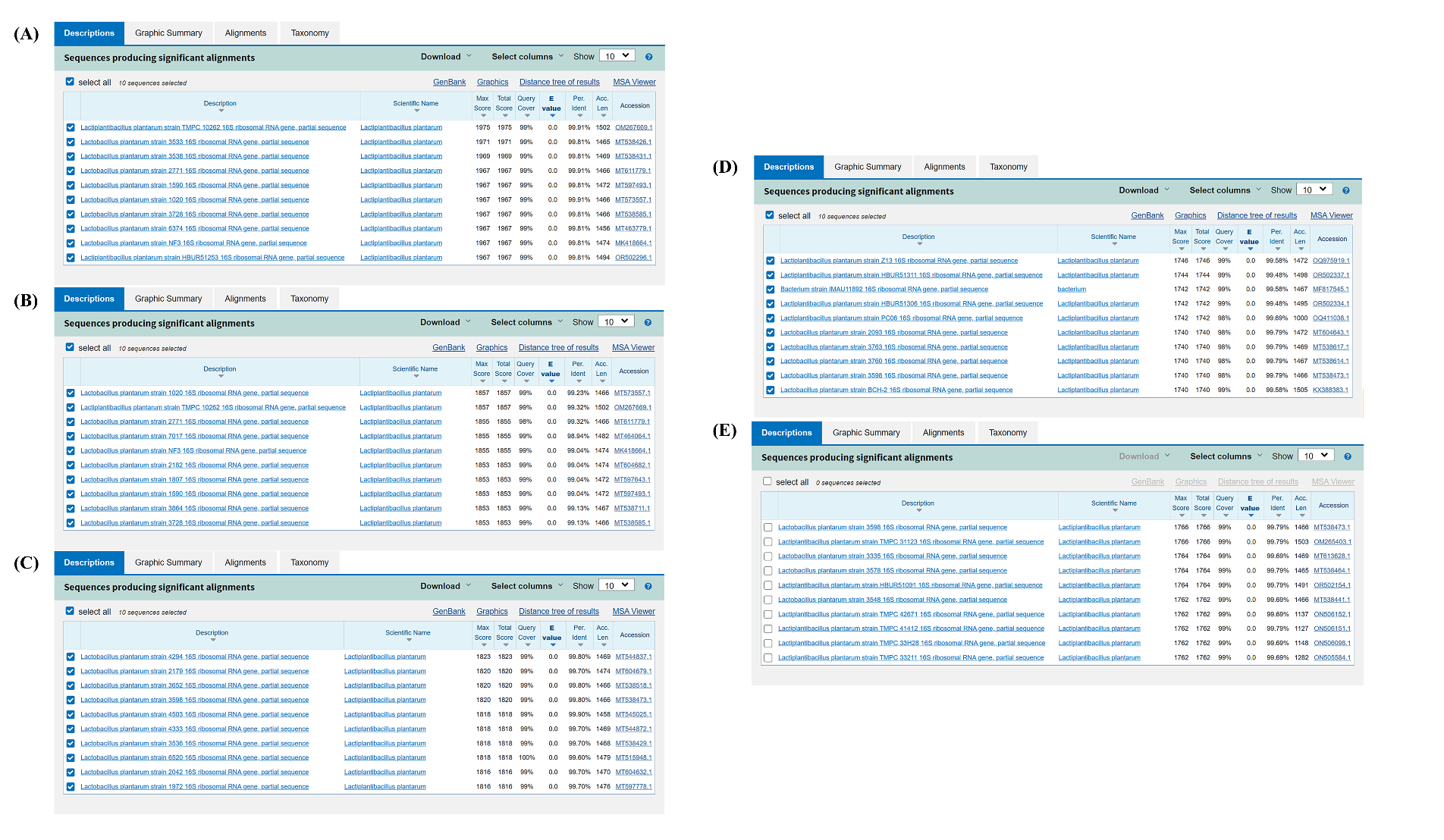


**Figure S1 The results of strain identification. (A)** ***L. plantarum* FWG097; (B) *L. plantarum* AJOP098; (C) *L. plantarum* TBP126; (D) *L. plantarum* WH137; (E) *L. plantarum* SRP140.**


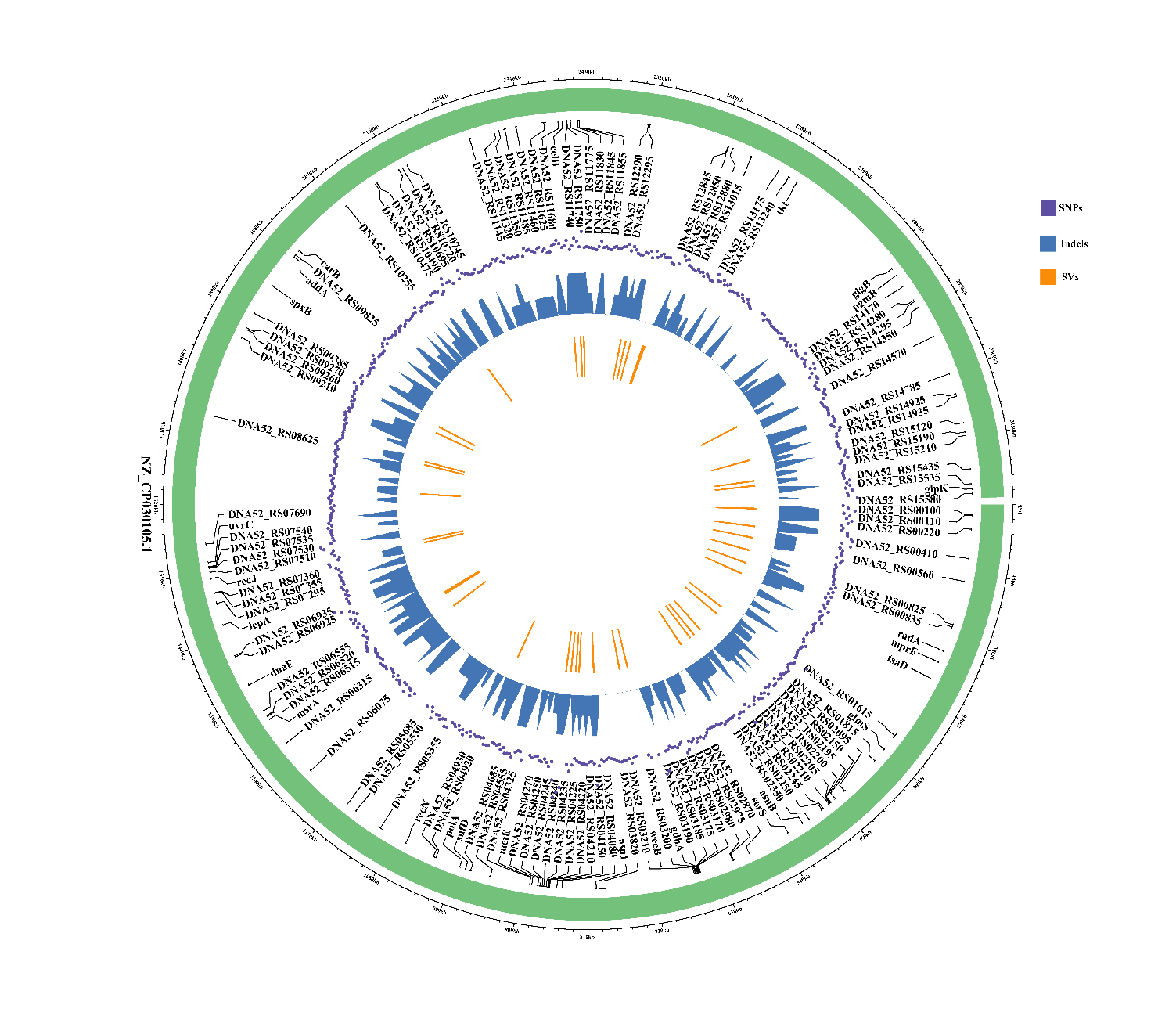


**Figure S2 The circos diagram of SNPs, Indels and SVs shared by 5 strains of *L. plantarum***


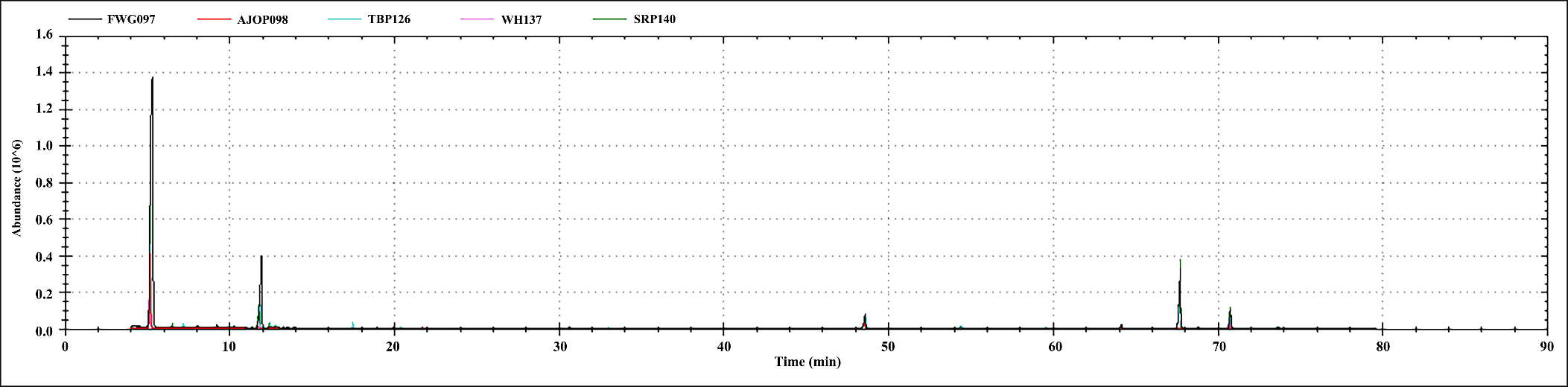


**Figure S3** **Total ion chromatography of intracellular metabolites of *L. plantarum***


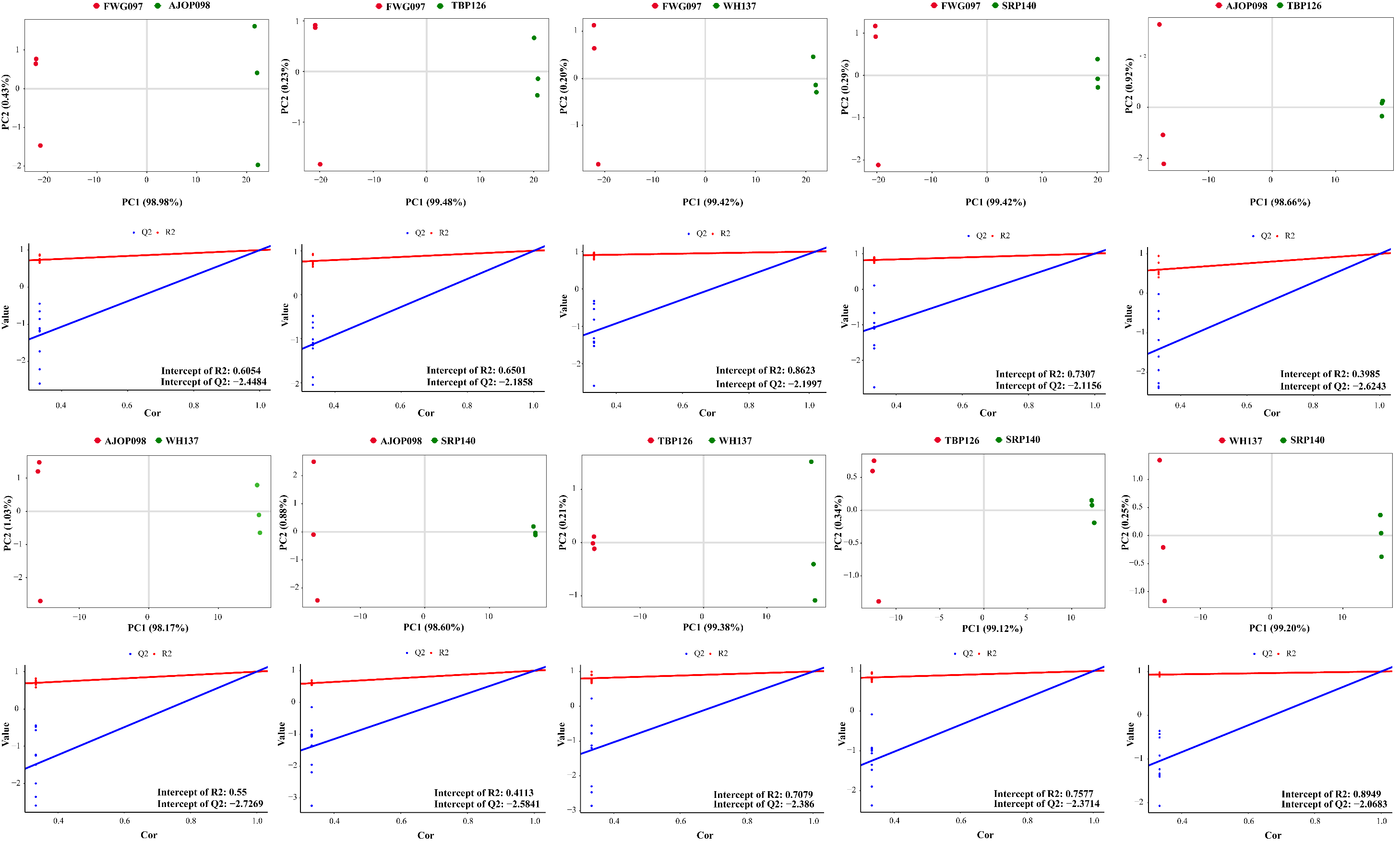


**Figure S4** **PLS-DA analysis.**
